# The Role of Transcription Factor PPAR-γ in the Pathogenesis of Psoriasis, Skin Cells, and Immune Cells

**DOI:** 10.3390/ijms23179708

**Published:** 2022-08-26

**Authors:** Vladimir V. Sobolev, Ekaterina Tchepourina, Irina M. Korsunskaya, Natalia A. Geppe, Svetlana N. Chebysheva, Anna G. Soboleva, Alexandre Mezentsev

**Affiliations:** 1 Center for Theoretical Problems in Physico-Chemical Pharmacology, Russian Academy of Sciences, Moscow 109029, Russia; 2 NF Filatov Clinical Institute of Children’s Health at I.M. Sechenov First MSMU, Moscow 119435, Russia; 3 Scientific Research Institute of Human Morphology, 3 Tsurupa Street, Moscow 117418, Russia

**Keywords:** PPAR-γ, psoriasis, skin, immune cells

## Abstract

The peroxisome proliferator-activated receptor PPAR-γ is one of three PPAR nuclear receptors that act as ligand-activated transcription factors. In immune cells, the skin, and other organs, PPAR-γ regulates lipid, glucose, and amino acid metabolism. The receptor translates nutritional, pharmacological, and metabolic stimuli into the changes in gene expression. The activation of PPAR-γ promotes cell differentiation, reduces the proliferation rate, and modulates the immune response. In the skin, PPARs also contribute to the functioning of the skin barrier. Since we know that the route from identification to the registration of drugs is long and expensive, PPAR-γ agonists already approved for other diseases may also represent a high interest for psoriasis. In this review, we discuss the role of PPAR-γ in the activation, differentiation, and proliferation of skin and immune cells affected by psoriasis and in contributing to the pathogenesis of the disease. We also evaluate whether the agonists of PPAR-γ may become one of the therapeutic options to suppress the inflammatory response in lesional psoriatic skin and decrease the influence of comorbidities associated with psoriasis.

## 1. Introduction

Psoriasis is a chronic inflammatory skin disorder characterized by the accumulation of red, scaly plaques on the skin. In some patients, the disease deforms and damages the joints and nails. It may also target oral mucosa. In the skin, psoriasis causes hyperproliferation and altered differentiation of epidermal keratinocytes. These result in thickening and structural remodeling of the epidermis. In the dermis, psoriasis promotes microvascular proliferation with the formation of abnormal dilated and tortuous capillaries [1]. Compared to healthy individuals, psoriasis patients have an increased risk of developing comorbidities, including cardiovascular disease, hypertension, obesity, and diabetes mellitus (rev. in [2,3]). A higher risk of cardiovascular, metabolic, and other disorders suggest that psoriasis is systemic, and in addition to the skin, it also damages other tissues and organs.

PPAR-γ (*NR1C3*) is one of three known isotypes of PPAR receptors. Two other isotypes, namely PPAR-α (*NR1C1*) and -β/δ (*NR1C2*), are highly homologous to PPAR-γ. All three proteins have the same domain composition (rev. in [4]). They also recognize the same sequence on the DNA (see below). However, they have different expression patterns across tissues and organs. Moreover, their specificity to the ligands differs. In addition, they play distinct roles in intracellular signaling and metabolism. In this regard, the activation of PPARs may produce a different outcome (rev. in [5,6]).

The expression of PPAR-γ is higher in white adipose tissue, the large intestine, the spleen, lymphoid tissue, and bone marrow. Its expression level in the kidney, heart, small intestine, ovary, testis, liver, transitional epithelium of the bladder, and epidermal keratinocytes is moderate. In skeletal muscles, the pancreas, and the brain, the expression of PPAR-γ is at a low level (rev. in [7]). To date, we know about six different transcripts of PPAR-γ, namely PPAR-γ1, -γ1Δ5, -γ2, -γ2Δ5, -γ3, and -γ4. All of them are the products of the same gene. They appear due to alternative splicing and the usage of alternative promoters (rev. in [8]). Although their encoding mRNAs are different, PPAR-γ1, -γ3, and -γ4 have the same amino acid composition. For this reason, we will refer to them as PPAR-γ1. PPAR-γ2 has an additional sequence at the N-terminus (28 amino acids in mice or 30 amino acids in humans). PPAR-γ1Δ5 and -γ2Δ5 are both missing the fifth exon that encodes the ligand-binding domain. Respectively, ligands of PPAR-γ recognize PPAR-γ1 and PPAR-γ2 transcripts. However, they are not capable of interacting with PPAR-γ1Δ5 and -γ2Δ5. The mentioned isoforms have different expression patterns. Adipose tissue and the intestine predominantly express PPAR-γ2. The other tissues, including the immune cells, mainly express PPAR-γ1 [9]. The endogenous expression of PPAR-γ1Δ5 and -γ2Δ5 positively correlates with the body mass index (BMI) in overweight or obese people. Their levels also increase in patients with type 2 diabetes. Since both Δ5 isoforms are incapable of interacting with ligands, they repress the ligand-dependent biological effects of PPAR-γ (see below). For instance, they impair adipogenesis, glucose, and lipid metabolism, which contribute to the development of metabolic syndrome [10].

In psoriasis, the activation of PPAR-γ modulates the inflammatory response by reducing the expression (see below) and downregulating the genes of adhesion molecules [11]. Moreover, the activation of PPAR-γ also inhibits the differentiation of Th CD4^+^ cells to Th_17_ cells. According to preliminary results, PPAR-γ controls ~150 genes directly associated with the disease [12].

## 2. Interaction of PPAR-γ with Ligands

Many biological effects of PPAR-γ require its interaction with ligands. As in the case of other receptors, the binding of PPAR-γ to a ligand forms a ligand–receptor complex. Ligands that bind to PPAR-γ can be endogenous, natural, or synthetic. The ligands can be specific to PPAR-γ or interact with other receptors. In addition, they can be either agonists or antagonists. The former activate PPAR-γ. The latter abolish its biological effects. In the absence of agonists, PPAR-γ forms protein complexes with transcriptional repressors (e.g., nuclear receptor corepressor (NCoR), silencing mediator of retinoid and thyroid receptors (SMRT), and TNF-induced protein 3 interacting protein 1 (TNIP1)) [13,14]. The named repressors interact with chromatin-modifying enzymes that exhibit histone deacetylase activity (e.g., HDAC3) [15]. By deacetylating the histones, the repressors make the chromatin inaccessible to other transcription factors, actively repressing the transcription (rev. in [16]).

Binding an agonist causes conformational changes in the molecule of PPAR-γ. These conformational changes lead to the appearance of new surfaces capable of interacting with transcriptional activators. They also cause dissociation of transcriptional repressors and their exchange for transcriptional activators, such as the members of the steroid receptor co-activator (SRC) family (rev. in [17]). These transcriptional activators exhibit a histone acetyltransferase activity. Histone acetylation by acetyltransferases makes the chromatin accessible for transcription. In other words, if PPAR-γ does not bind an agonist, it binds repressors that may potentially block the transcription of PPAR-γ controlled genes. Otherwise, if PPAR-γ binds an agonist, it “trades” repressors for activators and becomes capable of activating the transcription.

Most of the endogenous agonists of PPAR-γ are either polyunsaturated fatty acids (PUFAs) or their derivatives. These compounds are a part of fatty acid metabolism [18]. In turn, receptors for the named compounds, including PPAR-γ, are often referred to as sensors of fatty acids [19]. Several fatty acid metabolites (e.g., leukotriene B4, 8S-hydroxyeicosatetraenoic acid, and 12(R)-hydroxy-5,8,14-eicosatrienoic acid) that act through PPARs exert anti-inflammatory properties [20,21]. Many endogenous agonists bind to PPAR-γ in a micromolar concentration. However, some endogenous agonists, namely J-series prostaglandins (e.g., 15-deoxy-Δ 12,14 prostaglandin J2 (PGJ2)) and 9- and 13-HODE (9- and 13-hydroxyoctadecadienoic acid) have a high affinity to PPAR-γ. For this reason, many authors refer to them as physiological agonists. At the same time, their concentration in the cell is too low and, as believed, it is not enough to activate the receptor [22]. Moreover, the binding of PGJ2 to PPAR-γ is irreversible. In this case, the receptor–ligand complex remains constitutively activated until its disposal. In addition, PPAR-γ is capable of interacting with ligands of other receptors. For instance, ajulemic acid, the agonist of the cannabinoid receptor CB2, is capable of binding to and activating PPAR-γ. Acting on PPAR-γ, this compound prevents the development of fibrosis in mouse models of bleomycin-induced systemic sclerosis [23,24]. In this regard, blocking either CB2 or PPAR-γ inhibits their antifibrotic activity [23].

**Figure 1:**
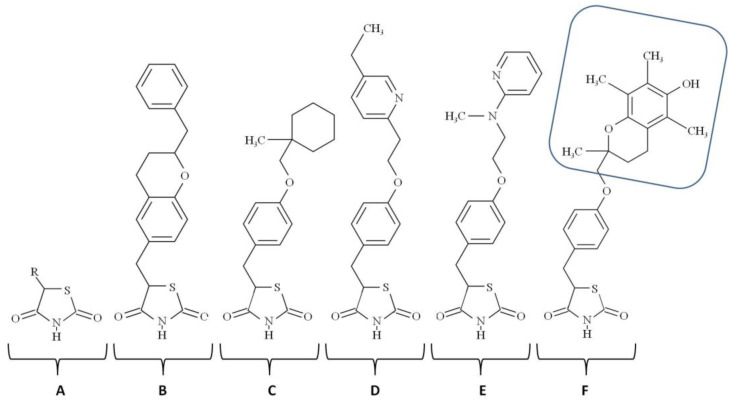
Chemical structures of the thiazolidinediones: A—thiazolidinedion; B—englitazone; C—ciglitazone; D—pioglitazone; E—rosiglitazone, F—troglitazone (the tocopherol group of troglitazone is shown inside the rounded blue rectangle).

The most well-studied synthetic agonists of PPAR-γ are thiazolidinediones (TZDs). Their molecules contain a five-atom C_3_NS ring (Figure 1). Compared to the most endogenous agonists of PPAR-γ, TZDs have a higher affinity to the receptor and are less crossreactive. The EC_50_ values of the most studied TZDs are 13.39 Μm for englitazone, 3 μM for ciglitazone, 906 nM for pioglitazone, 302 nM for rosiglitazone, and 0.55 μM for troglitazone. TZDs are well-known antidiabetic medicines. In patients with type 2 diabetes, they improve the sensitivity of the patients to insulin. They also reduce lipids and systemic inflammatory markers in blood plasma. Moreover, TZDs exert cardiovascular benefits that prevent the restenosis of the coronary artery and lower the blood pressure [25]. Some biological effects of TZDs can be beneficial for patients with chronic inflammatory disorders, including psoriasis. TZDs downregulate proinflammatory cytokines [26]. They also promote the nonproliferative phenotype of vascular smooth muscle cells VSMC [27] and modulate the Th_1_/Th_2_ transition in T lymphocytes [28]. In the skin, TZDs exhibit antiproliferative, anti-inflammatory, and immunomodulatory effects. Although TZDs are efficient activators of PPAR-γ, some of their beneficial effects do not require an interaction with the receptor. For instance, they inhibit the CDK5-mediated phosphorylation of PPAR-γ at Ser_273_ [29] and Ca^2+^ and K^+^ channels in a receptor-independent manner [30]. Although some receptor-independent biological effects of TZDs are beneficial for patients, these effects are nonspecific.

To distinguish between specific and nonspecific biological effects of TZDs, experimenters verify whether TZD and non-TZD agonists produce the same physiological outcome. If the result is achievable with the only type of agonists, the effect is nonspecific. Alternatively, researchers attempt to reproduce the desired effect in PPAR-γ deficient cells or animals. If the result is evident despite PPAR-γ deficiency, it is PPAR-γ-independent, i.e., nonspecific. Troglitazone (Figure 1) was the first TZD approved for the market, but it was later withdrawn due to hepatotoxicity. It contains the head group of α-tocopherol (vitamin E). Some other TZDs (e.g., BP-1003) contain the antioxidant α-lipoic acid/thioctic, 1,2-dithiolane pentanoic acid attached to benzoxy-TZD. The third TZDs, rosiglitazone and pioglitazone, do not contain biosimilar groups. In other words, they do not resemble derivatives of other bioactive compounds.

Like TZDs that exhibit nonspecific (PPAR-γ-independent) effects, the other drugs may interact with PPAR-γ as their secondary target. For instance, the primary target of nonsteroidal anti-inflammatory drugs (NSAIDs) is cycloxygenase 2/COX2/PTGS2. However, some NSAIDs also produce nonspecific effects by interacting with PPAR-γ [31]. Their interaction with PPAR-γ occurs at pharmacologically relevant concentrations. According to the results of an X-ray analysis, diclofenac and ibuprofen bind to PPAR-γ similarly to the other agonists. In the case of indomethacin and sulindac sulfide, PPAR-γ binds two of their molecules. The experiments that followed confirmed the ability of NSAIDs to regulate PPAR-γ-dependent target genes and promote adipogenesis in cultured 3T3-L1 fibroblasts. The authors also showed that knocking PPAR-γ disabled the named biological activities in the cells. For this reason, they concluded that NSAIDs produced their beneficial effects via their interaction with PPAR-γ. However, these effects were nonspecific to their primary (canonical) target (COX2/PTGS2).

The group of agonists known as selective modulators of PPAR activity (SPPARMs) consists of compounds that interact with a comparable affinity to either two or all three PPARs. For reference, the term “receptor modulators” applies to all compounds capable of binding to the receptor and changing its activity. For instance, BP-1003 (EC_50_~16 nM) and BP-1017 activate as PPAR-γ (EC_50_~66 nM) and PPAR-α (EC_50_~5 μM) [32]. These compounds exhibit the biological effects previously reported for TZDs. For instance, they inhibit the proliferation of human keratinocytes and suppress the production of IL2 by human peripheral lymphocytes. Some PPAR-γ agonists originate from natural sources. For instance, the plant *Astragalus propinquus* is the source of astragaloside IV. Although this compound is a potent activator of PPAR-γ [33], it also modulates the other signaling pathways [34]. The roots of *Astragali Radix* are in demand in traditional Chinese medicine for their potent anti-inflammatory and antitumorigenic effects. The authors of the cited papers explained some of these effects as the result of the ability of astragaloside IV to activate PPAR-γ. Unlike PPAR-γ agonists that activate the receptor, PPAR-γ *antagonists* (e.g., bexarotene, 2-phenylamino pyrimidine, and N-biphenylmethylindole derivatives) inhibit the activation of the receptor and downregulate the genes controlled by PPAR-γ. In clinical practice, the antagonists of PPAR-γ improve the uptake of glucose. They also stimulate osteogenesis and inhibit adipogenesis (rev. in [35]). The most frequently used antagonist of PPAR-γ is GW9662.

The ligand-binding site of PPAR-γ is a large and deep pocket (LDP) located inside its ligand-binding domain. Due to its huge volume, the LDP docks compounds of different sizes and chemical compositions. Interacting with ligands, PPAR-γ adapts itself to various conformations. When various ligands bind to the distinct subregions of LDP, they produce different physiological outcomes [36]. Moreover, some ligands of PPAR-γ (e.g., MRL-24 and SR1664) have an additional (allosteric) binding site [37]. Presumably, the binding of a second molecule follows the occupation of the regular binding site. Similar to the canonical activation discussed above, the allosteric regulation of PPAR-γ activates the receptor.

*In summary*, PPAR-γ is capable of interacting with various ligands. Some ligands (agonists) activate the receptor. The others (antagonists) interfere with its biological effects. In general, there are three groups of ligands: endogenous, natural, and synthetic. Some of them, including TZDs, not only serve PPAR as ligands, but also have other PPAR-independent biological activities. PPAR-γ interacts with ligands of other receptors. It also binds to pharmaceuticals targeting nonreceptor proteins (e.g., NSAIDs). In addition, PPAR-γ has a second ligand-binding site that binds selected ligands. Their interaction produces an additive effect on the activity of the receptor. Further investigations of ligands in various metabolic and inflammatory pathways will provide insight into their potential therapeutic effects in chronic inflammatory diseases such as psoriasis.

## 3. Heteromerization of PPAR-γ with RXR

**Figure 2:**
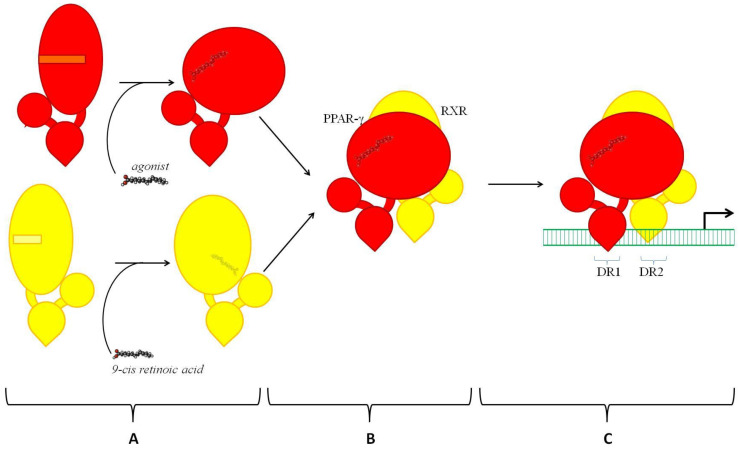
Transactivation of PPAR-γ. A—Interactions of agonist with PPAR-γ (shown in red) and 9-cis retinoic acid with RXR (shown in yellow). The activation of PPAR-γ and RXR by their ligands causes conformational changes. These changes cause dissociation of protein repressors and recruitment of activators (not shown). B—Heterodimerization of PPAR-γ and RXR. C—Binding of the heterodimer RXR-PPAR-γ to PPRE on the DNA. DR1 and DR2 are two halves of PPRE separated by a single nucleotide (N).

PPAR-γ exercises its gene-regulatory potential via transactivation and transrepression. Although both require an interaction of PPAR-γ with other transcription factors, they produce the opposite effect on transcription. Transactivation (Figure 2) promotes transcription. Otherwise, transrepression (see below) prevents it. To become capable of binding to DNA, PPAR-γ must heterodimerize with another ligand-activated nuclear receptor known as retinoid X receptor (RXR). All three known isotypes of RXR, namely RXR-α, -β, and -γ, are capable of interacting with PPAR-γ and forming productive complexes. Although each isotype has several isoforms due to alternative splicing, they share ligands. In addition, their heterodimers with PPAR-γ recognize the same DNA sequence (see below). On the other hand, RXRs have different expression patterns [38]. For instance, the most prevalent RXR isoform in epidermal keratinocytes, RXR-α, occupies 90% of active PPRE sites in the skin [39]. If the specific ligand 9-cis retinoic acid activates RXR, the interaction of RXR and PPAR-γ becomes stronger and facilitates the transcription, producing a synergistic effect [40,41]. However, the result of heterodimerization of PPAR-γ and RXR still depends on the activation of PPAR-γ. The ligandless PPAR-γ does not experience the necessary conformational changes. It does not exchange repressors for activators and, being in an inactive state, PPAR-γ disables the transcription.

In any state and composition (activated or not), the heterodimer (PPAR-γ–RXR) is a functional transcription factor. On the DNA, it binds to the specific site known as the peroxisome proliferator response element (PPRE). In the genome, many PPREs are present at 5′-flanking promoter regions of target genes. However, some PPREs can be a part of distant enhancer elements [42]. A typical PPRE represents the conserved DNA sequence AGGTCANAGGTCA. This sequence consists of two hexameric half-sites of AGGTCA separated by a single nucleotide (N). Although N can be any nucleotide, it is often adenine [43]. In turn, the two half-sites of PPRE are called DR1 and DR2. In addition, we must mention the homodimerization of RXR because the homodimers of RXR compete with PPAR-γ–RXR for the binding to PPRE [44]. Since RXR and PPAR-γ require different ligands for their activation, RXR-RXR and PPAR-γ–RXR produce differential effects on the transcription with different strengths. The choice between the named hetero- and homodimers is a choice between two sets of activators controlling the gene expression and their availability in the cell.

*In summary*, as a part of the heterodimer with RXR, PPAR-γ may either activate or repress transcription depending on its binding to an agonist. If PPAR-γ is bound to an agonist, PPAR-γ-RXR will induce it. Contrarily, it will repress it. Its influence on gene expression also depends on the competition with RXR-RXR and the availability of the specific coactivators.

## 4. Crosstalk of PPAR-γ with Other Transcription Factors

To date, genomewide binding profiles of PPAR-γ are available for different types of cells, including fibroblasts and immune cells [45,46]. The human genome contains several thousand active PPAR binding sites. Some of these sites are the parts of distant enhancer elements and introns. The others reside in proximal promoters of genes. According to the data obtained via a chromatin immunoprecipitation assay, the expression patterns of PPAR-γ are tissue-specific. Moreover, cells of the same type picked from different anatomical locations also have unique active PPREs [45,46,47]. A computer analysis performed in our lab demonstrated that the transcription factor PPAR-γ is a part of a regulatory network that unites several transcription factors, namely STAT3, FOXP3, NFκB, AHR, GATA3, HIF1A, FOXO1, and FOSL1 [48,49]. These transcription factors regulate each other at transcriptional and post-transcriptional levels [12]. Some of them, such as FOXO1, bind and repress the promoters of *PPARG*, which are responsible for the expression of γ1 and γ2 isoforms [50]. The others (e.g., the p65/RELA subunit of NFκB) lose their transcriptional activity via the formation of inhibitory complexes with PPAR-γ. In some cases, the crosstalk of two transcription factors is more complicated. For instance, the transcriptional factor FOSL1 also suppresses *PPARG* [51]. On the other hand, TZDs induce *Fosl1* during differentiation of 3T3-L1 cells [52], and the overexpression of a double negative form (*Pparg*) suppresses the inducible expression of *Fosl1* [53].

Considering psoriasis as a polygenic multifactorial disorder, we propose that the revealed network is decentralized [49]. In other words, none of the tested transcription factors, including PPAR-γ, play the role of a master key that connects its separate hubs and prevents it from falling apart. The named transcription factors have different competencies. For instance, the transcription factor PPAR-γ appears upstream of a few others, such as NFκB and AP1. In contrast, it can be under the control of FOXO1. According to our preliminary estimate [49,54], the discovered network regulates several hundred genes involved in psoriasis. About 90% of genes involved in the network are controlled in a combinatorial manner by several transcription factors. By changing the expression of growth factors and cytokines that modulate the differentiation of immune cells, the networked transcription factors regulate the intensity of the immune response. Moreover, they produce various biological effects on proliferation, differentiation, migration, and activation of immune and skin cells (see below).

## 5. Transrepression

**Figure 3:**
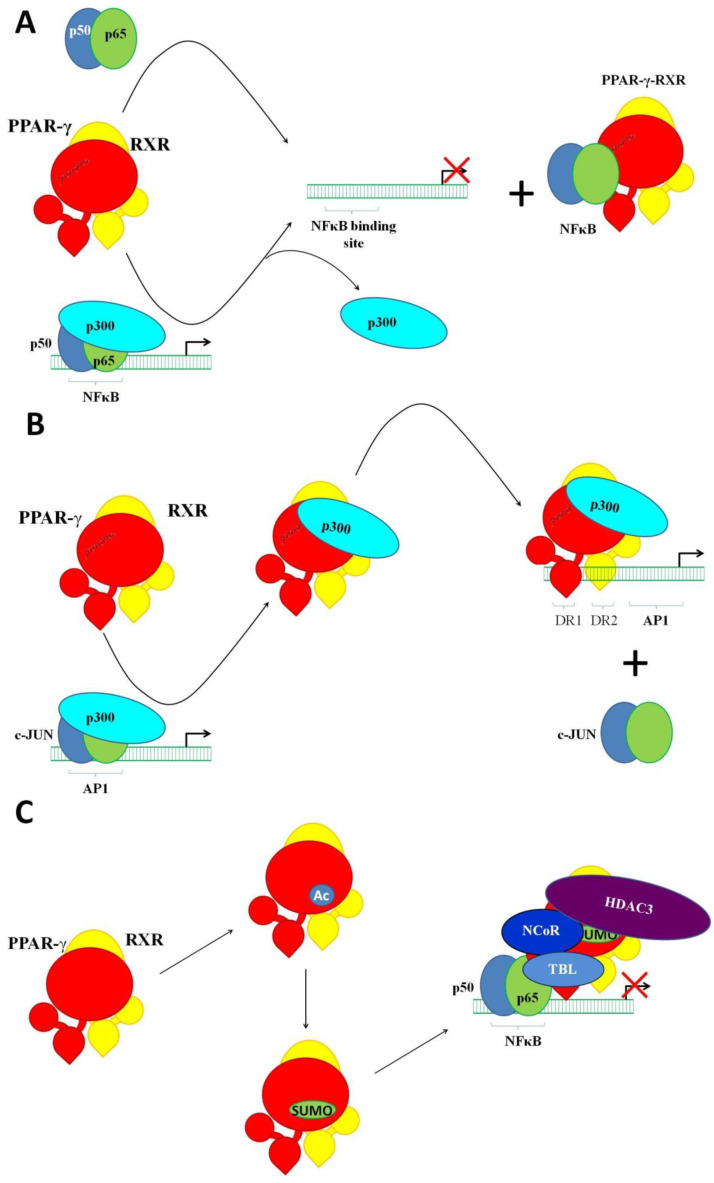
Transrepression by PPAR-γ. (**A**) The interaction of PPAR-γ with the RELA/p65 subunit of NFκB (tethering). The ligand-activated heterodimer of PPAR-γ and RXR replaces the transcriptional activator p300 in the complex, with the transcriptional factor NFκB attaching itself to the subunit p65. (**B**) The competition of PPAR-γ–p300 and AP1–p300 for binding to the promoter of *CCND1.* The ligand-activated heterodimer of PPAR-γ and RXR forcedly substitutes AP1 in its complex with p300. (**C**) SUMOlated PPAR-γ stabilizes the repressor complex NCoR–HDAC3–TBL bound to a gene promoter.

As we already mentioned above, PPAR-γ interacts with other transcription factors, including NFκB, AP1, NFAT, and STAT6, causing transrepression; i.e., silencing the transcription. Transrepression follows the activation of PPAR-γ with an agonist. There are at least two main mechanisms of transrepression, which are referred to as tethering and squelching. In the case of tethering, PPAR-γ attaches itself to another transcription factor, forming an inhibitory complex [55]. As a result, the targeted transcription factor loses the ability to activate transcription. Moreover, it does not matter whether the targeted transcription factor is already bound to the DNA. In turn, squelching is a sequestering of the regulatory proteins necessary for its activity. An example of tethering is the interaction of PPAR-γ with the RELA/p65 subunit of NFκB (Figure 3A). Their interaction prevents the binding of RELA to the DNA. It also promotes the export of RELA to the cytoplasm for degradation [56,57]. Moreover, PPAR-γ upregulates the transcription of IκBα, the major endogenous inhibitor of NFκB [58]. In addition, some agonists of PPAR-γ activate MAPKs in a PPAR-γ-independent manner, promoting the phosphorylation of the PPAR-γ. Then, the phosphorylated PPAR-γ binds to the complex of NFκB/IκB, preventing the phosphorylation and subsequent dissociation of IκB [59]. An example of squelching (Figure 3B) is the competition of PPAR-γ–p300 and AP1–p300 for binding to the promoter of *CCND1* [60]. Another example of squelching is a PPAR-γ-induced stabilization of the repressor complex NCoR–HDAC3–TBL that is already bound to a gene promoter (Figure 3C). In a stress situation, a specific external signal such as LPS triggers a signaling mechanism, disrupting the repressor complex and enabling the transcription. However, this is preventable by the activation of PPAR-γ with an agonist. The agonist-activated PPAR-γ undergoes conformational changes. They facilitate the site-specific SUMOylation of PPAR-γ at Lys_367_. After that, PPAR-γ gains the ability to interact with the repressor complex. Their interaction stabilizes the complex, preventing its dissociation from the DNA [61].

Similar to NFκB, PPAR-γ forms inhibitory complexes with AP1 [62], preventing the transcription of AP1 target genes associated with inflammation (adhesion molecules, cytokines, and metalloproteinases) and cell proliferation. Since these complexes do not interact with the DNA, their formation suppresses AP1 target genes (rev. in [63,64]). Likewise, the interaction of PPAR-γ with the nuclear factor of activated T cells (NFAT) inhibits the proliferation of T cells because it reduces the transcription of NFAT target genes. Briefly, the transcription factor NFAT induces *IL2* [65,66] and *IL4* [67]. However, the inhibitory complexes of NFAT and PPAR-γ effectively silence these genes because they cannot bind to the DNA. In addition, PPAR-y inhibits the differentiation of CD4^+^ T cells to Th_17_ cells controlled by the transcription factor ROR-γt [68]. When interacting with STAT3, PPAR-γ disables its binding to the *RORC* promoter. The latter reduces the expression of *ROR-γt* in CD4^+^ T cells, preventing the translation of *RORC* mRNA to ROR-γt, and inhibits their differentiation into Th_17_ cells [69]. In addition, PPAR-γ interacts with the repressor protein SMRT, which resides in the promoter area [70]. Their interaction stabilizes SMRT and disables transcription. Contrarily, a lack of PPAR-γ promotes the differentiation of Th_17_ cells [70].

*In summary*, transrepression caused by counteraction of PPAR-γ with other transcription factors is a major molecular mechanism that modulates the growth and differentiation of cells. It produces physicochemical and immunological stresses. In this regard, managing the level of PPAR-γ would represent a helpful tool to restore the functionality of cells affected by diseases such as psoriasis.

## 6. Tissue-Specific Regulation of PPAR-γ-Controlled Genes

The tissue-specific regulation of gene expression depends on the cooperation of various transcription factors [64]. Their interaction makes it possible for PPAR-γ to control different sets of genes in different types of cells. In mouse *macrophages*, PPAR-γ colocalizes on the DNA with the hematopoietic transcription factor PU.1. According to a chromatin immunoprecipitation analysis [47], both transcription factors reside in the areas of open chromatin near the sets of genes specifically expressed in macrophages. Contrarily, these genes are silenced in adipocytes because adipocytes do not express PU.1.

Szanto et al. [71] discovered a set of genes expressed in macrophages and dendritic cells. These genes are under the joint control of PPAR-γ and the transcription factor STAT6. STAT6 promotes the interaction of PPAR-γ with the DNA. In turn, the cooperation of PPAR-γ and STAT6 stimulates transcription. Notably, many sequences to which PPAR-γ binds are slightly different from the canonical PPREs. Their location is typically in distant enhancer elements, whereas their activation promotes the polarization of macrophages toward the anti-inflammatory (M2) phenotype (see below). In this regard, Szanto et al. proposed that the binding of STAT6 to the DNA facilitated the following binding of PPAR-γ with noncanonical PPREs [71]. They also provided experimental evidence of direct interaction between STAT6 and PPAR-γ.

In addition, Lefterova et al. [43] and Madsen et al. [72] discovered that genes implicated in adipogenesis are under joint control of PPAR-γ and C/EBPα. They and others [43,45] reported that 90% adipocyte genes required the binding of C/EBPα and C/EBPβ to the regulatory elements (promoters and enhancers) along with PPAR-γ in proximity of PPRE. By identifying thousands of new binding sites, they also substantially extended the list of genes dependent on PPAR-γ. They also revealed a cooperative nature of the molecular mechanism regulating their expression in adipocytes and preadipocytes. According to others [73], binding PPAR-γ to a regulatory element (intron, enhancer or promoter) follows the binding of C/EBP preassociated with repressors and histone deacetylases. Binding PPAR-γ causes the dissociation of the repressors, allowing the transcription to occur.

After reviewing the experimental data, we would like to mention that the two-factorial model proposed by the authors of the cited papers is not free of limitations. Primarily, it disregards the influence of other transcription factors occupying the same area [64]. Moreover, we would not exclude the situation when either transcription factor facilitates the loading of its neighbor [72]. The latter appears obvious, since both participants are potent to bind to their canonical binding sites independently. However, as we propose, these binding sites must be present in an area of open chromatin and made accessible to cooperating pairs of transcription factors by an unidentified third party.

*In summary*, the tissue-specific expression of PPAR-γ-dependent genes relies on PPAR-γ’s partnership with the so-called licensing transcription factors. The binding sites of both PPAR-γ and its partner are colocalized. Moreover, both transcription factors may even directly interact with each other. Preventing the licensing transcription factor from binding to the DNA also abolishes the binding of PPAR-γ and silences the target gene. Contrarily, if the licensing transcription factor has already occupied its binding site, it facilitates the binding of PPAR-γ to the DNA. This kind of cooperation is known as assisted loading. The diversity in the expression patterns that we see in different types of cells provides these cells with the specific abilities to control their differentiation and achieve the desired phenotype.

## 7. Post-Translational Modification of PPAR-γ

Like many other proteins, PPAR-γ undergoes post-translational modifications. After the protein folding, it interacts with specialized proteins that add modifying groups to the suitable amino acid residues. The addition of new chemical groups changes the functionality of PPAR-γ and adapts its properties to specific physiological conditions (rev. in [4,35]). In this section, we will discuss several post-translational modifications of PPAR-γ, namely phosphorylation, acetylation, ubiquitination, SUMOylation, and O-Glc-N-acylation.

### 7.1. Phosphorylation

Similar to other transcription factors, PPAR-γ is a phosphoprotein. Presently, we know about the phosphorylation of Tyr_78_, Ser_112_, Ser_133_, Ser_273_, and Thr_296_. In general, phosphorylation of some amino acid residues inhibits the transcriptional activity of PPAR-γ. According to the available data, at least four protein kinases, namely JNK, ERK1/2, CDK4, and CDK7, can phosphorylate Ser_112_ in PPAR-γ2 (rev. in [4]). The experiments on site-directed mutagenesis demonstrated that the replacement of serine by alanine (Ser_112_-Ala) modulated both the ligand-dependent and -independent effects of PPAR-γ. Expectedly, this mutation increased the transcriptional activity of PPAR-γ after its interaction with agonists [74].

A variety of stimuli such as epidermal growth factor (EGF), platelet-derived growth factor (PDGF), transforming growth factor β (TGFβ), and 12-O-tetradecanoyl-13-phorbol acetate (TPA) trigger the phosphorylation of PPAR-γ through the activation (phosphorylation) of ERKs and JNK. Contrarily, Ser_112_ is dephosphorylated by protein phosphatase 5 (PP5) [75], protein phosphatase Mg^2+^/Mn^2+^ dependent 1B (PPM1B) [76], and wild-type p53-induced phosphatase 1 (WIP1) [77]. In this respect, some authors hypothesized that phosphorylation-mediated inhibition of PPAR-γ-dependent transcriptional activity is a kind of emergency button that counteracts its activation with an agonist (rev. in [78]). At the same time, phosphorylation does not change protein stability. It also does not change the affinity of PPAR-γ to DNA, although it may lower the affinities of PPAR-γ to some ligands [74]. Phosphorylation of Ser_112_ enhances the interaction between PPAR-γ and the circadian clock protein period circadian regulator 2 (PER2) (rev. in [4]). Their interaction represses two groups of genes: the genes controlling adipogenesis and the genes predominantly expressed in brown adipose tissue (e.g., *CIDEA*, *ELOVL3*, and *UCP1*). Expectedly, the conventional knockout of PER2 produces the opposite effect; i.e., it increases their expression [79].

Protein kinases CDK5 and ERK1/2 also phosphorylate Ser_273_. As in the phosphorylation of Ser_112_, the phosphorylation of Ser_273_ inhibits the transcriptional activity of PPAR-γ [80]. On the other hand, it does not alter its adipogenic potential [81]. For reference, the level of phosphorylated Ser_273_ is higher in obese individuals. On the other hand, the activation of PPAR-γ by agonists counteracts the effects on transcription produced by the phosphorylation of Ser_273_. For instance, it improves the metabolic profiles of patients with impaired glucose tolerance. Some agonists of PPAR-γ (e.g., MRL-24, SR1664, and SR10171) block the phosphorylation of Ser_273_ without exhibiting a significant agonist activity. One day, these compounds may replace TZDs because by binding to PPAR-γ, they preserve it in the dephosphorylated form. Unlike TZDs, these compounds do not produce adverse effects such as fluid retention, bone loss, and weight gain [82,83]. In this respect, drugs blocking the phosphorylation of Ser_273_ should represent a better treatment option for patients with metabolic disorders.

The results of LC-MS/MS studies confirmed the phosphorylation of Ser_133_ and Thr_296_ [80]. According to the authors, ERK and MEK1/2 phosphorylate the first residue, whereas CDK5 phosphorylates the second. Another group provided evidence that proto-oncogene tyrosine-protein kinase SRC phosphorylates Tyr_78_ in vitro. Contrarily, protein-tyrosine phosphatase 1B/PTP-1B dephosphorylates it [84]. The introduction of mutant PPAR-γ (Tyr_78_-Phe) to PPAR-γ-deficient cells increases the expression of chemokines *CCRL2*, *CCL2*, *CCL5*, *CCL7*, *CCL9*, *CCL10*, *CCL11*, *CSF1*, *CSF2*, and *CXCL10* compared to the cells transfected with fully functional PPAR-γ. Moreover, it upregulates the expression of *IL6*, the endogenous inhibitor of matrix metalloproteinases (*TIMP1*), retinol-binding protein 4 (*RBP4*), and resistin (*RETN*).

### 7.2. SUMOylation

SUMOylation is a covalent attachment of the SUMO peptide to the target proteins by SUMO proteins. The molecule of PPAR-γ2 contains two SUMOylation consensus motifs around the residues Lys_107_ and Lys_395_ recognized by the SUMO1 protein [85,86,87]. A similar modification may occur with Lys_77_ and Lys_317_ [86]. In addition, the SUMO2 protein targets Lys_63_, Lys_94_, Lys_98_, and Lys_107_ [88]. For reference, SUMO1 knockout mice develop a metabolic phenotype because impaired SUMOylation changes the expression of PPAR-γ-controlled genes [89]. In turn, targeting Lys_395_ stabilizes the NCoR-containing repressive complex. Respectively, this post-translational modification causes suppression of PPAR-γ-controlled genes involved in the inflammatory response. In addition, there is cooperation between phosphorylation and SUMOylation. Yamashita et al. found that the phosphorylation of Ser_112_ promotes the SUMOylation of Lys_107_ [85] and suppresses the transcriptional activity of PPAR-γ. For instance, the SUMOylation of Lys_107_ suppresses the genes of several proinflammatory cytokines controlled by PPAR-γ due to the stabilization of the repressor complex NCoR–HDAC3–TBL [61]. Contrarily, a lack of SUMOylation results in the upregulation of various PPAR-γ target genes [90].

### 7.3. Acetylation

The molecule of PPAR-γ contains seven identified sites of acetylation: Lys_98_, Lys_107_, Lys_184_, Lys_185_, Lys_218_, Lys_268_, and Lys_293_. Binding of the TZD agonist rosiglitazone blocks the acetylation of Lys_268_ and Lys_293_ [91]. In turn, the acetylation of Lys_268_ and Lys_293_ promotes the interaction of PPAR-γ with the corepressor NCoR and suppresses the gene expression. Contrarily, their deacetylation facilitates the binding of the brown adipogenic activator PR domain containing 16 (PRDM16). The latter stimulates the browning of white adipose tissue and improves the sensitization of insulin. In addition, the acetylation of Lys_268_ and Lys_293_ influences the phosphorylation of Ser_273_ [91]. Selective deletion of NAD-dependent deacetylase sirtuin-1 deacetylase (Sirt1) lacks Lys_268_ and Lys_293_ in the acetylated form and causes dephosphorylation of Ser_273_ [92]. The roles of the other acetylation sites remain unclear.

### 7.4. Ubiquitination

PPAR-γ can be a target of canonical and atypical ubiquitination. Canonical ubiquitination marks PPAR-γ for degradation in proteasomes. Atypical ubiquitination by either neural precursor cell expressed developmentally downregulated 4 (NEDD4) [93] or tripartite motif-containing 23 (TRIM23) [94] stabilizes PPAR-γ, protecting it from the degradation.

### 7.5. O-Glc-N-acylation

Mass spectrometry and site-directed mutagenesis revealed the attaching of β-O-linked N-acetylglucosamine (O-GlcNAc) to Thr_84_ of PPAR-γ2 [95]. Treatment of cultured HeLa cells with the specific O-GlcNAcase (OGA) inhibitor 1,2-dideoxy-2′-propyl-α-d-glucopyranoso-[2,1-D]-Δ2′-thiazoline (NButGT) decreased the binding of PPAR-γ to PPRE in a luciferase reporter assay by 30%. It also delayed adipogenesis in 3T3-L1 cells. At the same time, the mutant Thr_84_Ala PPAR-γ was insensitive to the inhibitor.

*In summary*, post-translational modifications of PPAR-γ, namely phosphorylation, SUMOylation, ubiquitination, and O-GlcN-acylation, negatively regulate its transcriptional activity. In this regard, the phosphorylation of Ser_112_, Ser_273_, and Tyr_78_ suppresses the transactivation. The SUMOylation of Lys_107_ inhibits transcription by stabilizing the repressor complex NCoR–HDAC3–TBL in the promoter area. The acetylation of Lys_268_ and Lys_293_ has a dual effect. Mainly, it promotes the interaction of PPAR-γ with the corepressor NcoR; i.e., it decreases the gene expression. However, it also facilitates the dephosphorylation of Ser_273_ and the following transactivation. Regular ubiquitination marks PPAR-γ for degradation. In contrast, an atypical one rescues PPAR-γ from the degradation in proteasomes.

## 8. Ligand-Independent Stimulation of Macrophages

Cooperation with the transcription factor STAT6 that we briefly mentioned above is an example of the ligand-independent biological effects of PPAR-γ [96]. It occurs in monocytes that differentiate into type 2 (M2) macrophages. Briefly, the differentiation of monocytes to both M1 and M2 requires extracellular stimulation [96]. Their interaction with proinflammatory cytokines (e.g., TNF and INF-γ) and TLR ligands (e.g., LPS) triggers the differentiation to M1 macrophages. This type of macrophage exhibits proinflammatory activities. They are actively involved in phagocytosis and maintenance of the inflammatory response. Contrarily, the exposure of monocytes to either IL4 or IL13 promotes their differentiation to M2 macrophages. Unlike M1 macrophages, this type of macrophage expresses PPAR-γ. They are actively involved in wound healing and tissue repair. For instance, M2 macrophages intensively express the protein components of ECM (rev. in [97]) needed for resolving damaged epidermis and the repair of the skin barrier.

**Figure 4:**
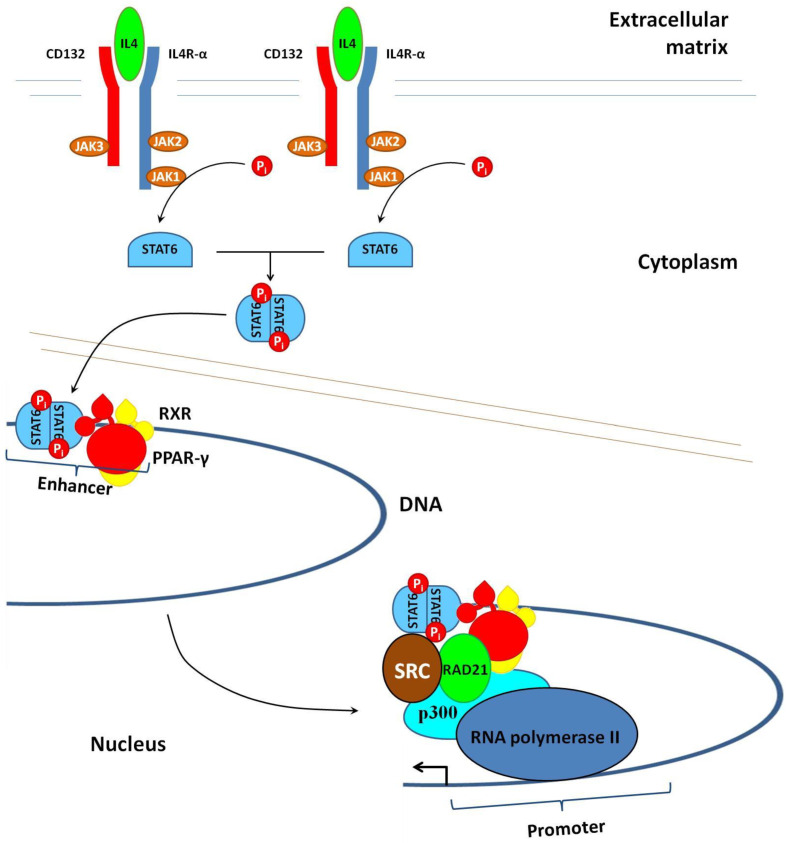
Transcriptional activation of the genes involved in polarization of macrophages toward the anti-inflammatory M2 phenotype. Binding of IL4 to the specific receptor (IL4R-α) triggers phosphorylation of the transcription factor STAT6 by JAK1. Phosphorylated STAT6 homodimerizes and then crosses to the nucleus, where it interacts with IL4-sensitive/RGS-insensitive enhancers of the DNA. This interaction recruits the heterodimer of agonist-free PPAR-γ and RXR, which are transcriptional activators. It also causes structural changes in the chromatin, making it accessible for RNA polymerase II and the transcription of genes.

The treatment of macrophages with IL4 or IL13 activates specific receptors such as IL4R-α (Figure 4). The activated receptor recruits the common γ chain (CD132), and after forming the heterodimer (IL4R-α–CD132), initiates protein phosphorylation by Janus kinases (JAKs). One of them, namely JAK1, phosphorylates STAT6. Phosphorylated STAT6 homodimerizes and translocates to the nucleus [98]. After the translocation, the homodimer of STAT6 binds to IL4-sensitive/RSG-insensitive enhancers. The first part of their name indicates that this class of enhancers is active only in IL4-stimulated cells. The second part of their name tells us that these enhancers remain inactive in the presence of rosiglitazone; i.e., their activation requires a ligand-free PPAR-γ. After the binding to the enhancer, STAT6 interacts with ligandless PPAR-γ. In turn, their interaction facilitates the recruitment of the transcriptional activators p300 and RAD21, which promote transcription [99].

The set of genes involved in the M2 polarization of macrophages includes arginase 1 (ARG1), CLEC10A/MGL1 [100], IL10, TGFβ, CD163, CD204, CD206, and MGL1/CD301a [101]. One of these proteins, the scavenger receptor CD163, is a specific biomarker of M2 macrophages [102]. By binding to the hemoglobin–haptoglobin complex, CD163 contributes to the utilization of extracellular iron during wound healing. In turn, the expression of CD163 is not detectable in M1-polarized macrophages.

The deficiency of PPAR-γ in macrophages slows down β-oxidation of fatty acids. For this reason, PPAR-γ-deficient macrophages cannot complete their transformation to the M2 phenotype [103]. Previous experiments in mice demonstrated that macrophage-specific deletion of PPAR-γ increased the ratio of the inflammatory M1 macrophages [103] and shifted the differentiation of CD^+^ Th cells toward the Th_1_ phenotype [104]. Similarly, the number of M1 cells increased in psoriasis patients compared to healthy control [105]. Since the M1 macrophages express the receptor of oxidized low-density lipoproteins (CD68), mediating their influx to the foam cells [106], shifting the differentiation of macrophages toward the M2 phenotype could partially explain the beneficial effects of PPAR-γ agonists in psoriasis patients with atherosclerosis.

## 9. The Role of PPAR-γ in Skin Metabolism

The prevalence of PPARs in tissues depends on their role in the metabolism of resident cells. In healthy epidermis, the prevalent form of PPAR is PPAR-β/δ because PPAR-β/δ plays a significant role in the maintenance of the skin permeability barrier and the biogenesis of lipids. The epidermal keratinocytes also express some PPAR-α and even less PPAR-γ [107]. In differentiating keratinocytes, the expression of PPAR-β/δ does not change. At the same time, the levels of PPAR-α and -γ mRNAs increase. Immunostaining for PPAR-γ in keratinocytes is visible in the nucleus and prenuclear region [108]. In the basal layer of healthy epidermis, the expression of PPAR-γ is almost undetectable [109]. During the terminal differentiation, the level of PPAR-γ increases 5-fold, peaking in the suprabasal layer [110,111]. The expression of PPAR-γ is also robust in the hair matrix keratinocytes, dermal papilla cells, the inner root sheath of the hair follicle [112], and sebocytes [113].

In the lesional psoriatic epidermis, the expression of PPAR-α and -γ is decreased compared to a healthy control. In contrast, the expression of PPARβ⁄δ increases due to the developing inflammatory response [114]. Psoriasis patients with multiple sclerosis, diabetes, and hypertension have significantly less PPAR-γ compared to the others [111]. There are also significant correlations of immunostained PPAR-γ in the skin and HDL (r = 0.376, *p* = 0.003), PASI (r = −0.591, *p* < 0.001), BMI (r = −0.312, *p* = 0.001), and blood glucose levels (r = −0.546, *p* < 0.001). In this regard, some authors [111] suggested that the reduction of PPAR-γ characterizes the metabolic state of psoriatic patients. Moreover, they proposed using PPAR-γ agonists as an adjuvant therapeutic tool to treat psoriasis patients with multiple sclerosis.

According to the studies performed on mice, either overexpression of PPAR-γ or its activation by agonists may potentially produce variable beneficial effects on the skin (see below). By shifting the balance between differentiation and proliferation toward differentiation, they normalize the terminal differentiation of epidermal keratinocytes and decrease their proliferation rate. The activation of PPAR-γ also modulates the biological effects of infiltrated immune cells and decreases the permeability of dermal microcapillaries. It also reduces the inflammatory response and improves the functioning of the skin barrier. Treatment of animals and cultured cells with agonists of PPAR-γ (troglitazone, rosiglitazone, pioglitazone, and BP-1107) decreases the proliferation rate of epidermal keratinocytes [108,115,116]. The antiproliferative effect is fully reversible and removes the used TZD from the culture medium [115,116]. The antiproliferative effects of PPAR-γ agonists are faster in recovering epidermis with a disrupted skin barrier [117]. For reference, TZDs activate the receptor at significantly *lower* concentrations than needed to inhibit cell proliferation. Thus, we are likely dealing with two separate phenomena. The first is the activation of PPAR-γ and the second is the activation of another PPAR by the same agonist with a lower affinity (rev. in [118]).

Downstream, the antiproliferative signaling of PPAR-γ agonists changes the expression of genes controlling the cell division. Due to transrepression, the expression of cyclins E/*CCNE1* and D1/*CCND1 decreases and their accumulation slows down* [119]. Contrarily, the genes of cyclin-dependent kinase inhibitors p21 (*CDKN1A*) and p27 (*CDKN1B*) become induced due to transactivation [120]. These changes caused by the activation of PPAR-γ lead to the cell cycle arrest in the G_1_ phase. In addition to the specific effects, TZDs also promote the phosphorylation of the eukaryotic initiation factor 2 (E2F). The phosphorylation of E2F prevents its binding to the DNA and reduces its transcriptional activity [121].

Topical treatment of healthy murine skin with agonists of PPAR-γ such as ciglitazone, troglitazone, and GI262570 accelerates the recovery of the disrupted skin barrier [117]. The treatment normalizes its functioning by improving the biosynthesis of cholesterol and ceramides [122]. The named agonists also induce the expression of cholesterol sulfotransferase type 2B1β (*SULT2B1β*) needed for the biosynthesis of cholesterol sulfate. The sulfotransferase plays a crucial role in desquamation of the cornified cells [123]. In turn, cholesterol sulfate induces the genes required to support the skin barrier. The agonists of PPAR-γ also induce the genes controlling the terminal differentiation of epidermal keratinocytes, namely involucrin (*IVL*), loricrin (*LOR*), transglutaminase-1 (*TGM1*), and filaggrin (*FLG*), in the skin of PPAR-γ-deficient mice (rev. in [124]). At the same time, they fail to produce similar changes in the skin of PPAR-β/δ- and RXR-α-deficient animals. Thus, these findings suggest that, unlike other PPARs, PPAR-γ can directly modulate the terminal differentiation of epidermal keratinocytes.

The agonists of PPAR-γ protect the skin from cutaneous inflammation. They suppress the genes of proinflammatory cytokines in resident skin cells. They also exhibit similar effects in invading immunocytes, vascular smooth muscle, and dendritic cells. The biological effect requires the activation of PPAR-γ, which, in turn, initiates the transrepression of NFκB and AP1 [125,126]. To be precise, the formation of inhibitory complexes between PPAR-γ and the named transcription factors suppress the genes of proinflammatory cytokines (IL6, IL8, IL12, IL21, IL23, and TNF). It also downregulates the expression of cyclooxygenase-2/COX-2/PTGS2 [127].

When discussing the ability of PPAR-γ to inhibit the expression of proinflammatory cytokines, we must acknowledge that complete blocking of the transcription factor NFκB by its specific inhibitors produces the opposite effect. Primarily, it significantly increases the proliferation of epidermal keratinocytes (rev. in [128]). Moreover, grafting the skin cells expressing a dominant-negative mutant IκBα on immunocompromised mice produces psoriasis-like skin lesions [129]. In addition, knocking out IKKα not only blocks NFκB, but also causes thickening of the epidermis. It also results in hyperplasia and impairs the terminal differentiation of epidermal keratinocytes [130]. In addition, treatment of the skin with UV light and dithranol induces NFκB and produces antipsoriatic effects in the epidermis [131]. We presume that these findings suggest that transrepression does not block some forms of NFκB from binding to the DNA, whereas its complete disabling results in much harsher consequences. In addition to their effects on growth and differentiation, TZDs also suppress the motility of cultured epidermal keratinocytes of the basal layer during wound healing [115]. This biological effect has relevance for psoriasis because the disease causes a weakening of the intercellular contacts in the epidermis. In this regard, modulating the cell motility will interfere with spreading the immune cells across the skin.

The biological effects of PPAR-γ and its agonists are also evident in the other types of skin cells. The activator of PPAR-γ rosiglitazone impairs melanogenesis in melanocytes [132]. Another PPAR-γ agonist, ciglitazone, induces the apoptosis of melanocytes in a dose-dependent manner [133]. Contrarily, treatment of cultured melanocytes with GW9662, the antagonist of PPAR-γ, stimulates their differentiation [134]. In addition, PPAR-γ agonists may influence the motility of melanocytes. According to Denkins et al., dietary ω-3 polyunsaturated fatty acids (PUFAs) such as eicosapentaenoic acid (EPA) and docosahexaenoic acid (DHA) decreased the motility of cultured 70W cells. At the same time, ω-6 PUFAs such as arachidonic acid produced the opposite effect [135].

In the sebaceous glands, the expression of PPAR-γ significantly increases during puberty [136]. PPAR-γ is also abundantly expressed by skin adipocytes, playing a crucial role in their differentiation [137]. Overexpression of PPAR-γ in fibroblasts decreases their expression of adhesion molecules ICAM1 and VCAM1, preventing the recruitment of leukocytes to the endothelial cells [138]. In vascular endothelial cells stimulated with 13-PMA, the agonists of PPAR-γ (15d-PGJ(2), ciglitazone, and troglitazone) reduce the expression of vascular cell adhesion molecule 1 (VCAM-1) and E-selectin [139]. In turn, the PPAR-γ agonist ciglitazone partially inhibits the production of chemokine C-C motif ligand 2 (CCL2) in the cells stimulated with C-reactive protein [140]. In addition, PPAR-γ modulates the INF-γ-induced expression of the chemokines CXCL9, -10, and -11, which subsequently reduces the chemotaxis of invading lymphocytes [141].

*In summary*, the level of PPAR-γ is low compared to other isotypes. In lesional skin, it is even less than in healthy skin. Among psoriasis patients, patients with multiple sclerosis express less PPAR-γ compared to the others. In mice and cultured cells, the agonists of PPAR-γ produce various biological effects that are potentially beneficial for psoriasis. They reduce the proliferation of epidermal keratinocytes and normalize their differentiation. They accelerate the restoration of the damaged skin barrier and suppress the genes of proinflammatory cytokines and adhesion molecules. In addition, they may potentially interfere with the infiltration of the skin by immune cells. Moreover, the agonists of PPAR-γ are efficient in other types of skin cells.

## 10. The Role of PPAR-γ in the Immune System

The migration of immune cells to lesional psoriatic skin causes their gradual accumulation in the plaques. It significantly changes the cellular composition of skin areas affected by the disease, altering the metabolism of resident skin cells and changing their appearance and functionality. In total, lesional skin may accumulate up to 20 million of the 28–30 million leukocytes in the human body [142]. In this regard, we would like to discuss the role of PPAR-γ in infiltrated immune cells due to their intensive crosstalk with resident skin cells.

A typical psoriatic infiltrate contains at least four major types of immune cells—lymphocytes, macrophages, dendritic cells, and neutrophils. The cellular composition of psoriatic infiltrate is not the same at various stages of the disease [143]. The ratio of macrophages reaches the maximum at the time when the disease exacerbates and psoriatic plaques continue to grow. The fraction of CD8^+^ Tc cells decreases with time. In contrast, the fractions of CD4^+^ T cells such as Th_1_, Th_17_, and Th_22_ increase even after the plaque growth slows down. The presence of neutrophils increases after the inflammatory process has already stabilized. The mentioned immune cells are preferably located in different parts of lesional skin because their mobility and the ability to interact with ECM are not the same [144,145]. CD4^+^ Th cells predominantly reside in the epidermis, whereas CD8^+^ T cells and neutrophils are in the dermis [146]. Macrophages accumulate in the dermal papillae and deeper dermis around the dilated superficial vessels. Moreover, they are also present in lymphohistiocytic infiltrates. In addition, large groups of immune cells remain in the papillary and reticular layers of the dermis [147].

Previous studies demonstrated that PPARs are highly expressed in macrophages [148], dendritic cells [149], neutrophils [150], B cells [151], and T cells [66]. In immune cells, PPAR-γ regulates their lipid metabolism and modulates the immune response. It influences their differentiation and proliferation. It controls the expression of cytokines and chemokines. Under certain conditions, the agonists of PPAR-γ induce the apoptosis of immune cells. The direct participation of PPAR-γ in various metabolic and signaling pathways makes it a potential molecular target for chronic inflammatory disorders such as psoriasis (rev. in [152]). In the following discussion, we will focus on the role of PPAR-γ in mediating the specific immune functions that are characteristic of certain types of immune cells altered in psoriasis.

## 11. Macrophages and Monocytes

In macrophages, PPAR-γ controls their polarization (see above). It plays a crucial role in phagocytosis and regulates the expression of proinflammatory cytokines. Being involved in the regulation of lipid metabolism, PPAR-γ controls the genes needed for the uptake of oxidized low-density lipoproteins (ox-LDL) and transportation of fatty acids (rev. in [152,153]). Activation of PPAR-γ by agonists modulates the metabolism of proinflammatory M1 macrophages [154,155]. The activated PPAR-γ stimulates the expression of cholesterol efflux-related genes—ATP binding cassette transporter A1 (ABCA1) [156,157] and acyl-coenzyme A:cholesterol acyltransferase (ABCG1) [157,158] through the liver X receptor α (LXR-α) pathway [19]. It accelerates the efflux of cholesterol from macrophages [156,158], inhibiting the growth of foam cells [156,159,160]. Contrarily, phosphorylation (i.e., counteracting the biological effects of agonist-activated PPAR-γ—see above) increases the expression of CD36- and SR-A1-related proteins and inhibits cholesterol efflux via ABCA1- and ABCG1-related proteins, promoting the transformation of M1 macrophages into foam cells [80,81,83].

By antagonizing with the transcription factors AP1 and NFκB, PPAR-γ exhibits anti-inflammatory activities. It downregulates the expression of the inducible nitric oxide synthase (*iNOS*) and gelatinase B (*MMP9*) [161]. Using the transrepression, PPAR-γ also downregulates the genes of chemokines and their receptors (e.g., *IL12*, *CD80*, *CXCL10*, and *CCL5*) [162]. Acting in a receptor-independent manner, the TZD agonists of PPAR-γ suppress the genes of proinflammatory cytokines (*TNF*, *IL6*, and *IL1β*) [163]. Moreover, PPAR-γ is crucial for phagocytosis [164]. Either ablation of PPAR-γ in cultured macrophages or its inhibition during the differentiation of monocytes to macrophages reduces their ability to engulf apoptotic cells [164,165]. Contrarily, treatment of nonprofessional phagocytes with PPAR-γ agonists improves their phagocytic abilities [166]. The latter occurs due to the transcriptional control of PPAR-γ over the participating cell-surface receptor molecules, complement receptors, and opsonins [165].

## 12. Dendritic Cells

PPAR-γ suppresses the maturation of dendritic cells and controls their production of retinoic acid [149,167]. As an immunomodulator, PPAR-γ influences the migration of dendritic cells. It also suppresses their expression of proinflammatory cytokines. In addition, PPAR-γ is involved in the presentation of lipid antigens by dendritic cells to inducible natural killer T cells (iNKTs) (rev. in [152]). When maturating, the dendritic cells dramatically change their expression of chemokine receptors. These changes stimulate their *migration* to the draining lymph nodes toward the gradient of CCL19 and CCL21 released from the lymphatic vessels (rev. in [168]). During the maturation, the activated PPAR-γ downregulates the surface receptor *CCR7*/*CD197* [169]. This receptor controls the signaling pathway that triggers the migration toward the named chemokines. The expression of CCR7 is also needed to induce the expression of MHC class I and MHC class II molecules, which are indispensable for the presentation of antigens [170]. Furthermore, the agonist-activated PPAR-γ influences the expression level of *CD1D*, a protein that presents lipid antigens to inducible natural killer T cells (iNKTs). The treatment of human monocyte-derived dendritic cells by PPAR-γ agonists indirectly stimulates *CD1D* via the induction of retinol and retinal metabolism enzymes [171]. These enzymes, namely retinol dehydrogenase 10 (RDH10) and retinaldehyde dehydrogenase type 2 (RALDH2), activate the biosynthesis of retinoic acids [172]. In turn, the accumulation of all-trans retinoic acid activates RAR-α, and RAR-α induces *CD1D* among its other target genes. By mediating the presentation of lipid antigen to iNKTs, *CD1D* promotes their activation [171,173].

In contributing to the maintenance of the Th_1_/Th_2_ balance, the activation of PPAR-γ in dendritic cells favors the development of type 2 immune response via suppression of Th_1_ cytokines. For instance, the agonists of PPAR-γ, such as 15d-PGJ2 and rosiglitazone, inhibit the production of IL12 in the cells stimulated with CD40 [174]. In addition, the same agonists of PPAR-γ suppress the production of CD80, CXCL10, and CCL5 involved in the recruitment of Th_1_ lymphocytes. The named agonists also downregulate *CCL3*, which participates in the recruitment and activation of polymorphonuclear leukocytes [175]. In addition, PPAR-γ accelerates the drug metabolism by inducing the gene of multidrug transporter *ABCG2* [176]. For this reason, the monocyte-derived dendritic cells expressing this gene gain an enhanced capacity to extrude xenobiotics. The latter improves cell survival and drug resistance during the therapy.

## 13. Langerhans Cells

PPAR-γ regulates the biological activities of Langerhans cells [177]. Increased expression of PPAR-γ affects their maturation and function mainly through the acceleration of lipid metabolism [171,178] and oxidation of fatty acids [179]. In turn, the activation of PPAR-γ with agonists promotes the differentiation of CD133^+^ hematopoietic progenitor cells to Langerhans cells and inhibits the differentiation of other dendritic cells. Treatment of the progenitor cells with rosiglitazone induces the expression of maturation-related antigens [180]. Most differentiating cells expressed CD207 (langerin), the specific biomarker of Langerhans cells. In addition, many cells contained Birbeck granules. Contrarily, the proinflammatory cytokines (e.g., TNF) inhibit the differentiation of Langerhans cells [178]. In addition, the PPAR-γ signaling pathway enhances immunogenicity and T-cell priming by Langerhans cells [181,182].

## 14. T Cells

Similar to other immune cells, PPAR-γ regulates the lipid metabolism of T cells. It also controls the expression of various proinflammatory cytokines. In addition, PPAR-γ plays a crucial role in their activation, which follows the recognition of antigens by T cells and causes rapid changes in their phenotype. In turn, it triggers multiple signaling pathways. It also stimulates their differentiation and proliferation. As a part of their activation, T cells shift from a quiescent state with a relatively low metabolic rate to a state with much higher metabolic demands [183]. During the activation, the cells become less reliant on oxidative metabolism. Instead, they activate anaerobic glycolysis (Warburg effect) [184]. The mentioned metabolic switch follows the stimulation of the T-cell receptor (Tcr). The stimulation of Tcr activates PPAR-γ and induces the genes that control glucose and fatty acid uptakes, namely *Glut1* and -*4*, *Ldlr*, *Lrp8*, *Scarb1*, and *Vldlr* [185,186]. In contrast, the expression of *Cd36* and uptake of ox-LDL by the cells remains unchanged [186].

By inhibiting NFAT, NFκB, and AP1 through transrepression (see above), PPAR-γ downregulates the proinflammatory cytokines [187]. Primarily, it reduces the production of IL2 required for the long-term proliferation of activated T cells. It also reduces their secretion of IL12 [174], IFN-γ [188], and TNF [189]. In turn, insufficient secretion impairs the effector functions of the other immune cells such as macrophages and natural killer cells [190]. This effect is receptor-dependent because the selective deletion of PPAR-γ in CD4^+^ T cells increases the biosynthesis of the named cytokines (e.g., [191]).

The activated CD4^+^ T cells differentiate into several subpopulations with different inflammatory and metabolic phenotypes, namely Th_1_, Th_2_, Th_17_, and T_reg_ [192]. The first three subsets of Th cells rely on glycolysis and intensively proliferate. Contrarily, the proliferation rate of inducible regulatory T cells (T_reg_) is lower. Unlike the other named subpopulations of T cells, T_reg_ cells rely on lipid oxidation (rev. in [193]). Expectedly, treatment of CD4^+^ T cells with various agonists of PPAR-γ reduces their proliferation. It also favors their differentiation toward T_reg_ [65,66]. Moreover, the agonists inhibit their differentiation toward Th_1_ [194] and Th_17_ [70] phenotypes. In addition, the activation of PPAR-γ by agonists makes T cells more susceptible to apoptosis [195]. Contrarily, deletion of PPAR-γ in CD4^+^ T cells reduces their apoptotic rate and restores their proliferation [191].

PPAR-γ plays a crucial role in the survival of T_reg_ cells [189]. The activation of PPAR-γ in naïve CD4^+^ T cells induces the transcription factor forkhead box P3 (FoxP3), promoting their differentiation toward T_reg_ [191,196]. A deficiency of PPAR-γ decreases the numbers of Foxp3^+^ CD4^+^ Th cells and increases the numbers of CD4^+^ IFN-γ^+^ cells. Consequently, T-cell-specific PPAR-γ deficiency in mice prevents the recruitment of T_reg_ cells to mesenteric lymph nodes. At the same time, the mutant T cells increase the expression of apoptosis-related genes [197]. In human natural killer cells, the agonists of PPAR-γ suppress their main biologic functions, which are cytolytic activity and the production of INF-γ. The former occurs in a ligand-independent manner in the cells treated with ciglitazone. The latter requires the activation of the receptor [198].

## 15. B Cells

The agonists of PPAR-γ stimulate the differentiation of B cells and promote their production of antibodies [199]. The underexpression of PPAR-γ in B cells significantly affects their sensitization. When comparing B cells of PPAR-γ^(+/−)^ mice and their normal littermates, Setoguchi et al. [200] discovered a spontaneous activation of mutant B cells. When testing the hypothesis that the downregulation of PPAR-γ might increase the predisposition of mice to autoimmune disorders, the authors found that PPAR-γ^(+/−)^ animals developed more severe symptoms of induced rheumatoid arthritis compared to the PPAR-γ^(+/+)^ control. According to another group [201], stimulation of PPAR-γ^(+/−)^ B cells with various agonists of PPAR-γ produced an enormous proliferative response. Moreover, the agonists downregulated the expression of antiapoptotic proteins, namely cellular inhibitor-of-apoptosis proteins 1 and 2 (*cLap1*/*Birc2* and *cLap2*/*Birc3*), X-chromosome-linked inhibitor-of-apoptosis protein (*Xiap*), and FLICE-inhibitory protein (*cFlip*/*Cflar*). At the same time, the treatment did not change the expression of the regulatory proteins Bcl-XL and Bcl2. The reduced expression of PPAR-γ in B cells was also responsible for their survival. Treatment of B cells with either 15d-PGJ2 or ciglitazone reduced their survival rate and induced their apoptosis [151]. In addition, the agonists of PPAR-γ exhibited anti-inflammatory activity, reducing the expression of adhesion molecules and cytokines (e.g., *ICAM-1*, *CXCL8*, and -*10*) [188].

## 16. Neutrophils

According to previous findings, PPAR-γ agonists reduce the infiltration of neutrophils in experimental model systems. In freshly prepared human neutrophils, it occurs after exposure to troglitazone or 15d-PGJ2. This effect is proportional to the concentration of the agonist. Moreover, it is reversed by the PPAR-γ antagonist GW9662. The chemotactic activity of neutrophils depends on the expression of PPAR-γ. For instance, cells overexpressing constitutively active PPAR-γ were less sensitive to chemoattractants than the control [202]. In addition, some agonists of PPAR-γ (e.g., pioglitazone) suppress the expression of *VCAM-1* and *CD11B*/*CD18* in activated neutrophils, impairing their interaction with endothelial cells [203]. In addition, TZDs are capable of inducing apoptosis in neutrophils [204]. Although the detailed mechanism behind this phenomenon remains unclear, some authors suggested that PTGS2 synthesized prostaglandins can reduce it [205]. In this respect, Gilroy et al. showed that neutrophils obtained from indomethacin-treated rats exhibited a lower apoptotic rate after stimulation with PGD2 and 15d-PGJ2 [204]. According to Brown et al. [206], human patients with less active PTGS2 and lower levels PGD2 were more likely to develop neutrophilia (a delayed clearance of neutrophils).

*In summary*, the role of PPAR-γ in immune cells is well studied (Table 1). The agonist-activated PPAR-γ reduces cell proliferation. PPAR-γ holds the balance between different subpopulations of T cells, dendritic cells, and macrophages. PPAR-γ exerts various anti-inflammatory activities. It downregulates the genes of proinflammatory cytokines, chemokines, and adhesion molecules. The agonist-activated PPAR-γ delays the differentiation of immune cells toward the inflammatory phenotypes. PPAR-γ inhibits their migration and their ability to penetrate blood vessels. For this reason, a careful examination of molecular mechanisms underlying the biological activities of PPAR-γ and PPAR-γ-independent effects produced by selected groups of ligands would be helpful for a more objective assessment of its clinical potential and minimizing the adverse effects.

## 17. Genetic Ablation of PPAR-γ in Mice

The conventional knockout of PPAR-γ results in embryonic lethality at E10.0–E10.5 due to severe abnormalities in epithelial differentiation of trophoblasts and failure of placental vascularization [209,210]. In embryogenesis, PPAR-γ is also crucial for proper formation and development of cardiac and adipose tissues [209]. In mouse chimeras comprised of PPAR-γ null and wild-type cells, the null epidermal keratinocytes can participate in the formation of the epidermis. This finding suggests that PPAR-γ is dispensable for the terminal differentiation of epidermal keratinocytes [211]. After the placental rescue, the surviving mice develop lipodystrophy and insulin resistance [212,213]. Their skin does not contain sebaceous glands [214]. After their birth, the pups experienced abnormalities in the development of their hair follicles. The authors observed a temporary delay in the morphogenesis of the hair follicles. They also showed a reduced expression of the differentiation markers and transcriptional regulators required for the normal development of the pups. In addition, a microarray analysis of skin samples revealed significant downregulation of genes characteristic of mature adipocytes, namely *Fabp4*, *Adipoq*, *Plin1*, and *Rbp4*. The interfollicular epidermis of null mice was dry with moderate white flaking. On day 17 after birth (P17), the skin experienced an infiltrate of immune cells that was mainly composed of macrophages and neutrophils. However, the level of Th cells was not different from the control. The histological analysis performed at P28 revealed hyperkeratosis and hyperplasia. The dermis was missing the intradermal adipocytes and sebaceous glands. The subcutaneous fat layer was also absent. The aging mice developed scarring alopecia and severe perifollicular inflammation. In addition, about 10% of the mice exhibited skin lesions.

In turn, a conditional deletion of PPAR-γ in the skeletal muscle leads to the development of glucose intolerance, hyperinsulinemia, and severe insulin resistance [215]. However, treating the mice with TZDs effectively reduced the harmful effects of a fat-enriched diet. For this reason, the authors concluded that TZDs might act in a PPAR-γ independent manner [216]. At the same time, the targeted deletion of PPAR-γ in the *adipose tissue* resulted in marked changes in adipose morphology: hypocellularity and hypertrophy. It also caused an elevation of free fatty acids in the plasma [217]. Moreover, the authors reported reduced levels of plasma leptin and adiponectin. Respectively, the mice developed hypertriglyceridemia and insulin resistance. Despite all these defects, the conditional deletion of PPAR-γ does not change the blood glucose. The mutation does not affect glucose or insulin tolerance and insulin-stimulated glucose uptake by the muscles. Like mice with a PPAR-γ deficiency in skeletal muscle, treatment with TZDs of mice with PPAR-γ deficiency in adipose tissue reverses the insulin resistance in the liver. As in the previous case, the authors explained this phenomenon as a PPAR-γ independent response to the TZD drugs [217].

Deletion of PPAR-γ in macrophages impairs their phagocytosis. It also affects their polarization toward the M2 phenotype [103]. The mutation alters lipid handling and cholesterol efflux due to the downregulation of the related genes, namely *Abca1*, *Abcg1*, and *Apoe* [157,165]. It also leads to glucose intolerance and insulin resistance in skeletal muscle and liver. Despite these changes, the mice remain lean while consuming a regular diet [218]. A conditional knockout of PPAR-γ in T cells promotes the differentiation of CD4^+^ Th cells toward the Th_17_ phenotype. At the same time, it does not affect their differentiation into Th_1_, Th_2_, and T_reg_ cells [70]. Accordingly, the expression of RORγt in CD4^+^-PPAR-γ knockout T cells was significantly increased compared to the control (CD4^+^ T cells). The levels of the transcription factors T-bet, GATA-3, and FoxP3 that determine the differentiation of Th_1_, Th_2_, and T_reg_ cells, respectively, did not change. Compared to their littermates with the unaffected expression of PPAR-γ, the knockout mice were more susceptible to Th_17_-mediated autoimmune disorders. In an experimental model of induced experimental autoimmune encephalomyelitis, the knockout mice showed a significantly earlier onset and aggravated disease course. Moreover, the severity of the disease directly correlated with the total numbers of infiltrating CD4^+^ T cells in the CNS. In contrast, these mice did not show significant abnormalities in their antigen-specific IFN-γ-producing CD4^+^ T cells. The deficiency of PPAR-γ in B cells impaired their development and reduced the production of circulating antigen-specific antibodies [199]. The inability to produce germinal center B cells and plasma cells negatively correlated with the expression of MHC class II, *Bcl6*, and *Blimp1*. Moreover, the mice with a deficiency of PPAR-γ in B cells had lower titers of antigen-specific antibodies and low numbers of antigen-experienced antibody-secreting cells. On the other hand, these mice had no differences in B-cell population distribution within either the primary or secondary lymphoid organs during development.

The agonists of PPAR-γ have shown their efficiency in the experimental models of psoriasis. For instance, a topical application of the PPAR-γ agonist GED-0507-34L reduced the psoriasis-like skin lesions caused by the injection of healthy mice with IL21. In lesional skin of injected animals, the mentioned drug suppressed the accumulation of cellular infiltrate and prevented the development of epidermal hyperplasia. It also normalized the terminal differentiation of epidermal keratinocytes [127]. In the severe combined immunodeficient (SCID) mouse–human skin transplant model, the TZD agonist troglitazone inhibited the proliferation of epidermal keratinocytes. It also improved the histological characteristics of the transplanted lesional skin [108]. Likewise, topical application of agonist BP-1107 inhibited hyperplastic changes in the transplants of both psoriatic and healthy skin transplants and reduced their epidermal thickness [116].

*In summary*, studies of PPAR-γ null mice and mice with tissue-specific deficiency of PPAR-γ revealed that PPAR-γ plays a crucial role in various types of cells and organs in their differentiation and development. The performed studies confirmed that PPAR-γ is indispensable for adipogenesis. They also showed that a lack of PPAR-γ in trophoblasts leads to embryonic lethality. The mice deficient in PPAR-γ also developed a skin phenotype characterized by a chronic inflammatory response. Conditional knockouts of PPAR-γ in immune cells confirmed an active role of PPAR-γ in phagocytosis by macrophages and the production of antibodies by B cells. They also showed that PPAR-γ controls the differentiation of Th_17_ cells, which are the main driving force in the pathogenesis of psoriasis. Moreover, the agonists of PPAR-γ showed promising results in experimental models of psoriasis and psoriasis-like skin lesions caused by disease-associated cytokines. Some agonists of PPAR-γ also normalized the histological features of grafted psoriatic and healthy skin.

## 18. Clinical Studies

Due to their roles in lipid metabolism, cell proliferation, differentiation, and inflammatory response, PPARs directly participate in the pathogenesis of other skin disorders. Antiproliferative, anti-inflammatory, and prodifferentiation activities make them drugs of interest for atopic dermatitis and skin cancer. They can also be alternative treatment options for pigmentary diseases, scleroderma, and acne vulgaris (rev. in [219]).

The antipsoriatic effects of PPAR-γ agonists became evident in three psoriasis patients with type 2 diabetes [220]. When targeting diabetes with troglitazone, Pershadsingh et al. noticed a significant improvement in psoriasis. Later, they reported a similar effect in two of three lean and euglycaemic patients without insulin resistance [108]. The patients received systemic treatment with troglitazone at 400–600 mg/day for 10–12 weeks. The authors also discovered that troglitazone reduced hyperplasia and normalized other histological features of lesional skin. Later, Itoh et al. [221] presented a medical history of a 65-year-old man with nonalcoholic steatohepatitis, diabetes, and psoriasis. Initially, they treated the patient with ursodeoxycholic acid. However, they had to discontinue it because this regimen failed to control the patient’s hyperglycemia. After the authors started him on pioglitazone (150 mg/day), the patient achieved a complete remission of psoriasis.

For reference, Pershadsingh’s study continued with a series of ex vivo experiments on skin explants of psoriasis patients—three patients that we already mentioned above, two other responders to the therapy, and two healthy individuals [108]. First, the authors infused the samples with TZDs (troglitazone or ciglitazone) or PGJ2. After a short time, they found that each tested compound inhibited the growth of epidermal keratinocytes in a dose-dependent manner. The lesional epidermis infused with troglitazone even restored its normal histological appearance. However, the authors did not notice significant changes in the derma. Second, they took some samples of lesional skin and grafted them onto immunocompromised mice. After grafting, they treated the animals with oral troglitazone. Subsequently, the treatment normalized the histological characteristics of the transplants. Based on these findings, the authors linked the observed antipsoriatic effects to the activation of PPAR-γ.

Later, the same group discovered that oral treatment of psoriasis patients with rosiglitazone was not as effective as treatments with the other PPAR agonists. They performed two extensive double-blind, placebo-controlled studies on large cohorts of patients [222]. The authors treated the patients with 2, 4, or 8 mg/day of rosiglitazone. However, they did not find significant improvement in patients compared to the control group. The following pilot study of eight patients reported by Kuenzli and Saurat [223] confirmed their conclusion. The authors treated two groups of patients with either 0.5% rosiglitazone or the PPAR-β/δ-specific agonist tetradecylthioacetic acid over 30 days and did not find any apparent benefit of the drugs. On the other hand, replacing rosiglitazone with pioglitazone may significantly improve psoriasis in a patient [224]. Expectedly, this finding raised questions regarding the efficiency of percutaneous absorption to produce a desired therapeutic response and the systemic nature of mechanisms underlying the beneficial effects of PPAR-γ [225].

In this regard, the authors of the following studies would probably respond that the route of drug administration matters and favor oral therapy with TZDs over the topical application [108,220,226]. Moreover, the data from in vitro experiments suggested that some molecular mechanisms of TZDs on keratinocytes could be PPAR-γ-independent [109]. Pershadsingh et al. [227] reported that two psoriasis patients (one with and one without type 2 diabetes) demonstrated a marked improvement in plaque psoriasis after administering rosiglitazone at 8 mg/day after six months of therapy.

Like other antipsoriatic therapies, TZDs may not be efficient in some patients. For instance, Robertshaw and Friedmann [226,228] reported an improvement in four of five patients with moderate to severe psoriasis after a course of therapy with pioglitazone (30 mg daily). Their last patient withdrew from the study due to side effects, which were excessive weight gain and fluid retention. Despite a small number of participants, the results of this study are significant because the patients selected by the authors did not respond well to PUVA or/and systemic therapy. In other words, they represented potential consumers of new treatments that TZDs may become in the future.

The phase 2 double-blind, randomized trial of pioglitazone (15 or 30 mg/day for 10 weeks) vs. placebo reported by Shafiq et al. [229] demonstrated a significant improvement in 26 of 68 patients with regular clinical evaluation at weeks 2, 6, and 10. There was a dose-dependent improvement and a reduction in the median Psoriasis Area Severity Index (PASI) score in the pioglitazone-treated patients compared with the placebo group at the end of the tenth week. More than 40% of the patients achieved complete remission, compared with 12.5% of patients treated with a placebo. Moreover, the investigators did not find any evident changes at the earliest time point. At the end of the course, the PASI scores of patients treated with a placebo or 15 or 30 mg of *pioglitazone* were reduced by 22, 41, and 48%, respectively. In addition, they did not register any serious adverse events.

In turn, Hafez et al. [230] reported on the efficiency of oral pioglitazone in 48 psoriasis patients with moderate to severe psoriasis. The authors divided the patients into two groups of equal size. The patients received either pioglitazone at 30 mg/day or a placebo. After 12 weeks of treatment, 5 of 24 (21%) patients in the pioglitazone group compared to 1/24 (4%) patients in the placebo group achieved the primary outcome—PASI_75_. At the same time, the patients’ metabolic syndrome and insulin resistance did not improve significantly. Although changes in the PASI were significant in the pioglitazone group (*p* = 0.009), the mean PASI values showed no significant difference compared to the placebo (*p* = 0.067). The authors also reported several adverse effects in the pioglitazone group such as fluid retention and edema of the lower limbs with prevalence in female patients (*p* = 0.009). Some parameters such as elevated arterial blood pressure, headache, and dyspnea were more frequent in female patients despite no significant difference between both groups. Three pioglitazone and one placebo patient withdrew from the study due to cutaneous exacerbation.

Although tackling the metabolic syndrome by Hafez et al. was unsuccessful, the authors of a similar study came to a different conclusion. Performing a randomized open-label controlled trial of metformin and pioglitazone (reg. number: CTRI/2011/12/002252, India), Singh and Bhansali [231] revealed the beneficial effects of both drugs for metabolic syndrome in psoriasis patients. Like Hafez et al., the authors also recruited psoriasis patients with metabolic syndrome. However, their patients had mild to moderate psoriasis (PASI < 10%). They divided the participants into three groups and treated them with metformin (*n* = 16), pioglitazone (*n* = 23), or a placebo (*n* = 21). In addition, they supplied all patients with a standard topical 5% coal tar ointment. After finishing the study, Singh and Bhansali detected statistically significant differences in achieving a 75% reduction in the PASI and ESI in both the metformin and pioglitazone groups compared to the placebo group. At the same time, the effects of pioglitazone and metformin on the PASI were comparable (*p* = 0.528). According to the authors, 52.4 and 50% of their metformin and pioglitazone patients, respectively, improved their fasting plasma glucose and triglycerides. Moreover, treatment with pioglitazone significantly improved systolic and diastolic blood pressures, total cholesterol, and LDL cholesterol levels, which was not evident in Hafez’s study [230].

An open-label pilot study performed by Bongartz et al. [232] monitored the disease activity in 10 patients with active psoriatic arthritis. The authors treated the participants with a combination of pioglitazone (60 mg/day) and NSAIDs. In the end, the mean decrease in the PASI score was 38%. Moreover, two patients achieved a PASI_50_ response. Contrarily, ~25% of patients did not respond to the treatment. The authors also reported a statistically significant reduction in the average number of painful and/or swollen joints. According to their observations, the most significant improvements occurred between weeks 6 and 12. This observation suggested that three months of therapy would be a reasonable time to achieve sufficient clinical response. In addition, the authors noticed some side effects such as edema and weight gain.

In turn, Mittal et al. reported the results of randomized double-blind, placebo-controlled clinical trial (reg. number: NCT00395941, USA). The authors investigated a combination therapy of acitretin and pioglitazone in psoriasis [233]. For this trial, they selected patients that did not have diabetes or cardiovascular, metabolic, or other severe chronic disorders. After 12 weeks, they found that a combination of acitretin and pioglitazone reduced the PASI score by 64.2%. At the same time, the PASI scores of the patients treated with acitretin and placebo decreased by 51.7%. Based on their findings, Mittal et al. concluded that pioglitazone improved the action of acitretin. They also suggested that a combination with acitretin can become a relatively safe and effective option for psoriasis patients. In this trial, the authors reported an unrelated episode of acute myocardial infarction and referred to the other side effects as mild to moderate.

Likewise, a single-blinded randomized and controlled trial performed by Lajevardi et al. showed that pioglitazone enhanced the therapeutic effect of methotrexate patients with plaque-type psoriasis [234]. The authors treated two groups of 22 patients with either methotrexate only or methotrexate plus pioglitazone for 16 weeks. After the course, they discovered a reduction in the PASI scores in each group by 60.2 and 70.3%, respectively. They also noticed that patients treated with the combination of drugs were six times more likely to achieve the PASI_75_ mark than patients treated with methotrexate only. At the same time, they did not find any significant changes in the patients’ Dermatology Life Quality Index (DLQI) (63.6% vs. 56.9%).

In a similar randomized, open-labeled, parallel-group interventional study [235], Abidi et al. recruited 90 patients with moderate to severe psoriasis. They divided patients into three groups. The first group (*n* = 29) received methotrexate (7.5 mg/week), the second group (*n* = 30) received pioglitazone (15 mg/day), and the third group (*n* = 30) received both drugs for 12 weeks. The first and the last groups also received folic acid to counter the adverse effects of the methotrexate. In addition, the authors supplied the patients with topical therapy (clobetasol + salicylic acid). At the end of the study, the authors found that the combination of methotrexate and pioglitazone was more efficient than other treatments. At week 12, all patients in this group demonstrated a significant improvement. The dual therapy also improved the patients’ metabolic syndrome. The HDL levels were significantly higher in patients treated with pioglitazone (groups 2 and 3). These patients had significantly lower blood pressure and fasting plasma glucose. In addition, we would like to mention a better outcome achieved by Abidi et al. compared to Lajevardi et al. [234], which was presumably for two reasons. First, their study lasted 12 weeks instead of 10; second, it involved a double dose of pioglitazone.

A few years earlier, El-Gharabawy et al. [236] analyzed three groups of patients (*n* = 30 in each group) that were prescribed traditional antipsoriatic medicines (e.g., coal tar, vitamin D_3_ analogues, and corticosteroids), oral metformin (850 mg twice in a day), and pioglitazone (15 mg/day). In this study, the authors allowed the patients of the last two groups to use the traditional antipsoriatic medicines. At the completion of the study, they assessed the patients’ performance and compared them to control groups (healthy volunteers and patients that did not take medicines; 30 individuals each). The authors found that the numbers of CD4^+^ T cells significantly decreased in all three tested groups of patients. Moreover, the patients treated with pioglitazone showed a significant increase in CD8^+^ T cells compared to psoriasis patients without treatment. The authors also discovered that the levels of IL2, C-reactive protein (CRP), ceruloplasmin (CP), alanine, and aspartate aminotransferases significantly decreased in patients taking the medicines compared to both control groups. In this respect, we would like to acknowledge that the results obtained by El-Gharabawy et al. confirmed the previous findings. In this regard, we note that El-Sisi et al. [237] reported the ability of PPAR-γ agonists to inhibit the production of IL2 by T cells. The changes in the levels of CRP were consistent with a previous report published by Ferretti et al. [238]. They noticed that elevated levels of CRP indicated the progression of the inflammatory response even in patients with mild psoriasis. The changes in the level of CP confirmed the results of Shenoy et al. [239], who reported that the elevation of ceruloplasmin in psoriasis patients occurred in the acute phase of inflammatory response and could reflect its scavenging action on highly toxic hydroxyl radicals produced during the inflammatory process. The changes in the levels of aminotransferases were in agreement with the report by Razavizade et al. [240], who discovered similar changes in psoriasis patients treated with metformin. At the same time, El-Gharabawy et al. [236] did not assess changes in the PASI despite presenting a detailed biochemical analysis of the patients’ blood samples.

In a double-blinded randomized and controlled trial, Ghiasi et al. [241] investigated whether pioglitazone would improve phototherapy (Reg. Number: IRCT 16831, Iran). The authors divided 60 psoriasis patients eligible for phototherapy into two groups. Patients in the first group received pioglitazone (30 mg daily). Patients in the second group received a placebo. Moreover, both groups received 30 sessions of narrow-band UVB phototherapy. The initial dosage of phototherapy was 200 mJ/cm^2^. If the patients did not present with asymptomatic mild erythema, the authors increased the dosage by 40% per session. However, they did not exceed 3000 mJ/cm^2^. Starting with the 10th session, the average PASI of the pioglitazone group was significantly lower than that of the placebo group (*p* ~ 0.05). The PASI scores decreased from 20.9 ± 9.8 to 1.8 ± 1.4% and from 22 ± 8.5 to 4.4 ± 4%, respectively (*p* < 0.05). Moreover, 85% of pioglitazone and 40% of placebo patients achieved PASI_75_. At the same time, the authors did not detect significant changes in the patients’ white blood cell and platelet counts, triglycerides, LDL, and total cholesterol. They also did not mention any severe effects except a nonsignificant rise in fasting blood sugar and the HbA1C level in the placebo group. Based on their findings, Ghiasi et al. recommended pioglitazone to enhance the efficacy of phototherapy in eligible psoriasis patients.

**Figure 5:**
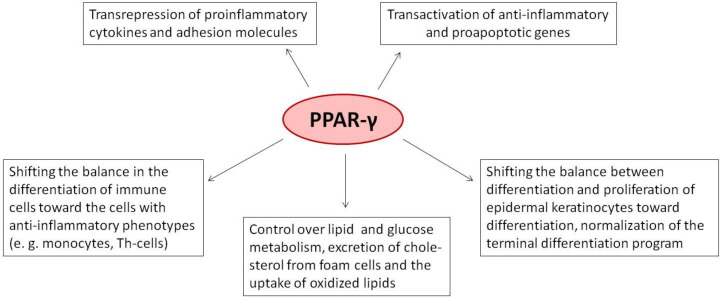
The role of PPAR-γ in the pathogenesis of psoriasis.

*In summary*, the agonists of PPAR-γ are efficient in normalizing the main morphologic features of psoriasis. Exhibiting several potentially beneficial effects, they may become a potential treatment option for the disease. Although TZDs are not completely replaceable and are not effective when applied topically, in some cases, they could be suitable for systemic therapy. According to the previously published results, the agonists of PPAR-γ can also produce an additive effect to the other treatments. The available data suggest that PPAR-γ exhibits potent anti-inflammatory activities (Figure 5). It suppresses the differentiation of immune cells with the proinflammatory phenotype. The transrepression caused by PPAR-γ suppresses the genes of proinflammatory cytokines and adhesion molecules. Due to transactivation, PPAR-γ activates the expression of anti-inflammatory and proapoptotic genes. In the skin, PPAR-γ decreases the proliferation of epidermal keratinocytes, normalizing their terminal differentiation program. By controlling lipid metabolism, PPAR-γ increases the biosynthesis of lipids. The latter, in turn, improves the functioning of the skin barrier. In addition, PPAR-γ stimulates the efflux of cholesterol from foam cells and reduces their uptake of ox-LDLs, delaying the progression of atherosclerosis in patients. Although regulators have approved TZDs for type 2 diabetes, their prescriptions are scarce [242] due to moderate effectiveness and evident adverse effects (see below).

## 19. Studies of Disease-Associated Polymorphisms

Single-nucleotide polymorphisms (SNPs) are genetic variations of a single nucleotide in a chromosome. These variations usually appear in at least 1% of the population. Like point mutations, polymorphisms may represent insertions, deletions, and substitutions at a specific position of the DNA. However, they are different from mutations because it is usually impossible to say what gene variation appeared earlier. Although we cannot define SNPs as mutations, we can identify ones associated with a disease of interest by comparing their frequencies in the patients’ genomes and the genomes of healthy volunteers using statistical analysis. In this regard, carrying a disease-associated SNP by a healthy individual may indicate a predisposition to the disease. The SNPs identified in a patient’s genome may suggest preferable treatment options. For instance, some SNPs discovered in the gene for PPAR-γ indicate a risk of obesity and severe insulin resistance [243]. The recommendations to patients with such SNPs will favor drugs that either do not provoke the named conditions or prevent their progression. According to some authors (see below), the other variations of PPAR-γ might indicate a higher risk of psoriasis.

In a case-control study performed by Mossner et al., the authors examined the possible associations between psoriasis and SNPs of PPAR-γ, namely rs1801282 (C34G, *Pro*12-*Ala*), and rs3856806 (C161T, *His*447-*His*) in Germans [244]. Comparing the frequencies of SNPs in patients (*n* = 192) and healthy individuals (*n* = 330), the authors did not find any significant differences associated with the disease (*p* = 0.6 and 0.78, respectively). On the other hand, they found it possible that other genetic alterations not examined in their paper may still play a role in the pathogenesis of psoriasis. In turn, Bowes et al. [245] verified a possible relevance for psoriatic arthritis of eight SNPs located in the coding area of the gene encoding PPAR-γ, including rs1801282. The authors analyzed 982 tissue samples donated by patients from Great Britain and Ireland. According to the obtained results, rs1801282 showed borderline evidence for association (*p* = 0.06).

In a similar study, Seleit et al. examined whether carrying Ala in the 12th codon of the PPAR-γ gene (rs1801282) may represent a risk for psoriasis in the Egyptian population [246]. The authors analyzed the genotypes that belonged to lean and obese psoriasis patients (*n* = 45) and an equal number of healthy volunteers with matched BMIs. They found that the homo- and heterozygous genotypes (Ala/Ala and Pro/Ala, respectively) increased the risk of psoriasis by 5.25- and 3.65-fold for the carriers (*p* = 0.01). Although earlier, Mossner et al. [244] did not find a significant risk of psoriasis among the individuals with rs1801282. Seleit et al. explained this contradiction as a result of differences in criteria used to select the participants, as well as ethnic differences between the Egyptian and German populations.

## 20. Adverse Effects

Like many other medications, PPAR-based therapies exhibit undesired adverse effects due to their simultaneous action on various cells and nonspecific off-target effects. The activation of PPAR-γ promotes adipogenesis and adipocyte maturation, leading to adipocyte hypertrophy. The patients gain weight (~5 kg in 3–5 years) because they accumulate fat and retain fluids [247]. The common side effect of PPAR-γ agonists is edema. In addition, TZDs increase the risk of cardiovascular events and bone fractures. We also acknowledge a higher risk of heart failure in patients with type 2 diabetes (rev. in [25]) and the cumulative effect of TZDs on hip fractures by 18% [248].

Pioglitazone and rosiglitazone are classified as pregnancy C category drugs, as animal models showed experimental evidence of growth retardation in mid to late gestation (rev. in [249]). In some countries, TZDs do not have the approval of local regulators. In 1997, manufacturers stopped the production of troglitazone due to a higher risk of hepatotoxicity. Pioglitazone was suspended in France and Germany (2011) due to a higher risk of bladder cancer. Rosiglitazone (Avandia), according to some reports (e.g., [250]), increases the risk of heart attack and death. These reports caused a withdrawal of rosiglitazone from the market in the UK, Spain, India, New Zealand, and South Africa. In other countries such as Russia, the mentioned drugs remain in use. In addition, we note the appearance of new safer and more efficient agonists of PPAR-γ, as well as dual and pan-PPAR agonists with a broader spectrum of action.

## 21. Future Perspectives of PPAR-γ Agonists in Psoriasis

The new generations of PPAR-γ agonists ought to preserve the beneficial characteristics of TZDs. They should enhance insulin sensitivity and decrease fatty acid oxidation. Moreover, they should cause less fluid retention and weight gain. We also anticipate that they will exhibit potent anti-inflammatory and antiproliferative effects on keratinocytes. Unlike TZDs, primarily troglitazone and pioglitazone, the new drugs must be less mutagenic and cytotoxic. Expectedly, some of these drugs can originate from natural sources. In this regard, we note amorfrutins from edible parts of *Glycyrrhiza foetida* and *Amorpha fruticosa* [251]. By activating PPAR-γ, amorfrutins significantly improve various metabolic parameters such as the resistance to insulin [252]. Similar to other agonists of PPAR-γ, amorfrutins exhibit anti-inflammatory activities [251]. However, they do not cause unwanted side effects such as increased fat storage and genotoxicity [253].

Alternatively, it would be reasonable to consider the agonists that selective blocking the phosphorylation of Ser_273_. Although the blockers of Ser_273_ do not exhibit robust agonist activity (e.g., MRL24, SR1664, and SR10171), they also do not produce significant adverse effects such as retention of fluid, bone loss, and weight gain [82,83]. In this respect, drugs blocking the phosphorylation of Ser_273_ should represent a better treatment option for patients with metabolic disorders than traditional TZDs.

Additionally, we note a group of compounds that serve as agonists to both PPARs-α and -γ; e.g., BP-1003 and -1017. Compared to TZDs, these drugs more efficiently inhibit the proliferation of human keratinocytes. They also suppress the production of IL2 by human peripheral lymphocytes. In an oxazolone-sensitized mouse model of allergic contact dermatitis (ACD), either oral or topical administration of BP-1017 reduces pinnal swelling. As we believe, these findings suggest that dual agonists have a clinical potential in hyperproliferative and inflammatory disorders such as psoriasis [32]. In this regard, a recently discovered non-TZD agonist of PPAR-γ, GED-0507-34L, binds to PPAR-γ with a relatively high affinity (EC50 ~ 500 nM) [127]. This agonist exhibits biological effects that are potentially beneficial for patients with chronic inflammatory disorders. First, GED-0507-34L reduces the inflammatory response by inhibiting the nuclear translocation of the transcription factor p65/NFκB with the consequent suppression of proinflammatory genes. Second, GED-0507-34L upregulates the expression of IκBα [127]. For reference, when targeting PPAR-γ, GED-0507-34L also suppresses PTGS2 in normal human epidermal keratinocytes and lymphocytes. It normalizes the proliferation rate of the cells and the expression of terminal differentiation markers altered by TNF. The latter is important, considering the involvement of TNF in the pathogenesis of psoriasis. In psoriasis-like skin lesions caused by IL21 in mice, a topical application of GED-0507-34L reduces the accumulation of cellular infiltrate and epidermal hyperplasia. In this regard, GED-0507-34L can potentially help patients with psoriasis and other inflammatory disorders.

Presently, GED-0507-34 L is under investigation for the treatment of inflammatory bowel and Crohn’s diseases [127], intestinal and colorectal fibrosis [254,255], and lichen planopilaris [256]. A phase I clinical study of psoriasis patients (Reg. Number: GED0507-PSO-01-12, USA) demonstrated that unlike TZDs, GED-0507-34L did not display serious side effects except redness and itching in treated areas (in 1 of 24 treated patients).

## 22. Conclusions

PPAR-γ modulates a diverse array of biological functions, and its agonists can become efficient therapeutic agents for various autoimmune disorders. Their potentially beneficial effects on keratinocytes and immunocytes make them promising drug candidates for psoriasis. Exhibiting diverse biological activities in both keratinocytes and immune cells, PPAR-γ represents an attractive object for understanding the nature of psoriasis and the development of new therapeutic approaches. Since the agonists of PPAR-γ act on the main histological features of psoriasis, they have clinical relevance. With advancing research and the development of newer agonists, the scope of PPARs in dermatological practice will increase further. However, the current knowledge about PPARs and certainly PPAR-γ agonists suggests that PPARs-targeting agents may not be very efficient in some patients. In this paper, we highlighted the role of PPAR-γ in immunity and inflammation. We discussed the functions of this receptor in the skin and immune cells. We also analyzed the role of PPAR-γ in the pathogenesis of the disease. As we believe, future studies will shed light on the other less-known aspects of the PPAR-γ signaling pathway and address existing safety concerns.

## Figures and Tables

**Table 1:** The role of PPAR-γ in immune cells.

Type of Cells	Function/Biological Effect of PPAR-γ	References
Macrophages	Promotes polarization of macrophages toward anti-inflammatory M2 phenotype	[148,149]
	Promotes phagocytosis of apoptotic cells	[158,159,160]
	Downregulates the genes of proinflammatory cytokines and their receptors	[156]
	Improves the transportation of fatty acids	[146,147]
	Induces the genes responsible for efflux of cholesterol	[150,151,152]
	Delays the growth of foam cells	[150,153,154]
Dendritic cells	Suppresses the maturation of dendritic cells	[163,164]
	Indirectly controls the biosynthesis of reinoic acid	[143,161,165,166]
	Influences the migration of dendritic cells	[207]
	Suppresses the expression of proinflammatory cytokines	[168,169]
	Promotes the presentation of lipid antigenes to iNKTs	[146,164,165]
	Accelerates the drug metabolism	[170]
Langerhans cells	Modulates the maturation of Langerhans cells	[173]
	Accelerates lipid metabolism	[167,174]
	Increases the oxidation of fatty acids	[175]
	Promotes the differentiation of CD133^+^ progenitor cells toward Langerhans cells	[176]
	Enhances immunogenicity and improves T-cell priming	[177,178]
T cells	Stimulates the uptake of glucose and fatty acids	[173,174]
	Suppresses the genes of proinlammatory cytokines	[179]
	Contributes to the activation of T cells	[185]
	Promotes the differentiation of CD4^+^ T cells to T_reg_	[177]
	Inhibits the differentiation of CD4^+^ T cells to Th1, Th2, and Th17 cells	[64,182]
	Protects T cells from apoptosis, reducing the expression of proapoptotic genes	[185]
	Improves the survival of T_reg_ cells	[177]
	Inhibits the production of IFN-γ	[186]
B cells	Stimulates the differentiation of B cells	[208]
	Activates the production of antibodies by B cells	[208]
	Controls the activation of B cells	[188]
	Improves the survival of B cells	[145]
	Controls the expression of proapoptotic genes	[199]
Neutrophils	Reduces the infiltration of neutrophils, impairing their interaction with endothelial cells of blood vessels	[191]
	Reduces the sensitivity of neutrophils to chemoattractants	[190]
	Accelerates the clearance of neutrophils	[192]

## Data Availability

Not applicable.
